# Intrathecal hydrophilic opioids for abdominal surgery: a meta-analysis, meta-regression, and trial sequential analysis

**DOI:** 10.1016/j.bja.2020.05.061

**Published:** 2020-07-11

**Authors:** Mark V. Koning, Markus Klimek, Koen Rijs, Robert J. Stolker, Michael A. Heesen

**Affiliations:** 1 Department of Anaesthesiology, Erasmus University Medical Centre, Rotterdam, the Netherlands; 2 Department of Anaesthesiology and Critical Care, Rijnstate Hospital, Arnhem, the Netherlands; 3 Department of Anaesthesiology, Kantonsspital Baden, Baden, Switzerland

**Keywords:** analgesics, enhanced recovery, intrathecal, laparoscopy, laparotomy, opioids, spinal injections

## Abstract

**Background:**

Intrathecal hydrophilic opioids decrease systemic opioid consumption after abdominal surgery and potentially facilitate enhanced recovery. A meta-analysis is needed to quantify associated risks and benefits.

**Methods:**

A systematic search was performed to find RCTs investigating intrathecal hydrophilic opioids in abdominal surgery. Caesarean section and continuous regional or neuraxial techniques were excluded. Several subgroup analyses were prespecified. A conventional meta-analysis, meta-regression, trial sequential analysis, and provision of GRADE scores were planned.

**Results:**

The search yielded 40 trials consisting of 2500 patients. A difference was detected in ‘i.v. morphine consumption’ at Day 1 {mean difference [MD] −18.4 mg, (95% confidence interval [CI]: −22.3 to −14.4)} and Day 2 (MD −25.5 mg [95% CI: −30.2 to −20.8]), pain scores at Day 1 in rest (MD −0.9 [95% CI: −1.1 to −0.7]) and during movement (MD −1.2 [95% CI: −1.6 to −0.8]), length of stay (MD −0.2 days [95% CI: −0.4 to −0.1]) and pruritus (relative risk 4.3 [95% CI: 2.5–7.5]) but not in nausea or sedation. A difference was detected for respiratory depression (odds ratio 5.5 [95% CI: 2.1–14.2]) but not when two small outlying studies were excluded (odds ratio 1.4 [95% CI: 0.4–5.2]). The level of evidence was graded as high for morphine consumption, in part because the required information size was reached.

**Conclusions:**

This study showed important opioid-sparing effects of intrathecal hydrophilic opioids. Our data suggest a dose-dependent relationship between the risk of respiratory depression and the dose of intrathecal opioids. Excluding two high-dose studies, intrathecal opioids have a comparable incidence of respiratory depression as the control group.

**Clinical trial registration:**

PROSPERO-registry: CRD42018090682.

**Editor's key points**

• In this meta-analysis of 40 studies (2500 subjects), the authors investigated the analgesia provided following abdominal surgery by the use of intrathecal hydrophilic opioids.
• They found that opioid consumption and pain scores were reduced when intrathecal hydrophilic opioids were used, while pruritus was increased. Late respiratory depression occurred more often, but not when lower doses were used.
• The findings imply that use of low-dose intrathecal hydrophilic opioids provides analgesic and opioid-sparing effects in abdominal surgery, and that side-effects are limited.
• This technique may complement enhanced recovery programs.

Enhanced recovery programs (ERPs) are accompanied by multiple recommendations, one of which is sufficient postoperative analgesia.^1^ A promising analgesic approach is the use of intrathecal hydrophilic opioids, which have been used for decades, and renewed interest was caused by a recent study that was able to show an enhanced recovery in abdominal surgery.^2^^,^^3^ Still, the risks and benefits need to be quantified before the widespread use in abdominal surgery can be advocated.

The benefits of intrathecal hydrophilic opioids, compared with i. v. administration, are believed to be caused by a higher potency and a prolonged action, because of a small distribution volume of the CSF and a slow diffusion, respectively.^4^ Used as a single bolus technique, intrathecal hydrophilic opioids have an i. v. opioid-sparing effect, facilitate mobilisation and—because of a lack of peripheral vasodilation—a restrictive fluid management can easily be achieved.^5^ These properties may lead to a faster recovery after abdominal surgery.

The risks, however, are pruritus, nausea, and late respiratory depression. Especially the fear for the latter has limited the use of intrathecal hydrophilic opioids. Meylan and colleagues ^6^ performed a meta-analysis regarding intrathecal morphine, and they found higher rates of pruritus and respiratory depression. However, that meta-analysis involved predominantly studies in cardiac surgery and a wide range of dosages were used. This limits the transfer of the found risks and benefits to abdominal surgery, which requires a meta-analysis of its own.

Therefore, we performed a meta-analysis to quantify the risks and benefits of intrathecal hydrophilic opioids. Our study had two goals: firstly, we set out to identify the studies published in the last decade in order to come to an updated evaluation of the benefits and risks of intrathecal morphine. Secondly, we focused on a particular patient group (i.e. abdominal surgery patients undergoing both open and laparoscopic procedures). Furthermore, in recent years trial sequential analysis (TSA) has emerged as a statistical technique that maintains the Type 1 error-rate in meta-analyses at a prespecified level, which contributes to the certainty of a conclusion in a meta-analysis.^7^ This technique was applied to the data obtained from trials on intrathecal hydrophilic opioids for abdominal surgery.

## Methods

Our meta-analysis was performed in accordance with the PRISMA statement.^8^ The meta-analysis was registered at PROSPERO with registration number CRD42018090682.

A systemic literature search was performed in December 2019. We searched the databases of Medline, Embase, CINAHL, LILACS, Cochrane CENTRAL, Web of Science, ClinicalTrials.gov, and Google Scholar. Filters or language restriction were not applied. The search combined terms for ‘intrathecal’, ‘hydrophilic opioid’, and ‘abdominal surgery (see Supplementary material). Morphine, hydromorphone, diamorphine, pethidine, and dihydromorphine were considered hydrophilic opiates. The search was managed with EndNote and duplicates were removed. Bibliographies of selected studies were also screened for studies of interest. The search included trial registers and these records were checked for completion and publication.

Inclusion and exclusion criteria were defined *a priori*, and only randomised trials were considered. The inclusion criteria were defined according to a PICO-search, in which the Patients were adults undergoing abdominal surgery, the Intervention was the administration of intrathecal hydrophilic opioids, with or without additives, such as local anaesthetics, the Comparator was analgesia without intrathecal hydrophilic opioids. The primary outcome measures were i. v. morphine-equivalents consumption at 24 and 48 h. The secondary outcome measures were: pain scores in rest and during movement at 24 and 48 h; time to fit for discharge; length of hospital stay; time to first analgesic request; intraoperative sufentanil-equivalent consumption; and incidence of nausea, pruritus, sedation, and respiratory depression.

Exclusion criteria were Caesarean section and the use of concomitant continuous regional anaesthesia or neuraxial anaesthesia.

Two authors (MVK and MK) screened the abstracts for eligible studies. Full texts of these studies were analysed, and data were extracted if the study was considered includable. The extracted data were authors, year of publication, type of surgery, details of intervention, details of control, postoperative analgesia, and urinary catheter management. If the mean and standard deviation were not reported in the paper, we derived the mean and standard deviation from the median and range using the formula by Hozo and colleagues.^9^ Morphine equivalents were calculated. The conversion factor for piritramide was 0.7,^10^ for papaveretum 0.665,^11^ for fentanyl 100,^12^ for pethidine 0.133,^13^ and for tramadol 0.1.^12^ The conversion factor to calculate fentanyl into sufentanil equivalents for intraoperative analgesia was 0.1.^14^ If multiple groups with intrathecal morphine were compared, we combined those groups and used the mean dose of intrathecal morphine. If a trial used multiple groups that could serve as control groups (i.e. without intrathecal hydrophilic opioids), the group with the control treatment most similar to the intervention group was used. The continuous outcome measures of such a study were the mean values of the groups and the largest standard deviation of the groups. Additions of events and patients were used for binary data.

The methodological quality of each study was evaluated by two authors (MVK and MH) based on the Cochrane Risk of Bias tool.^15^ This tool includes assessment of the risks of selection bias (random sequence generation, allocation concealment), performance bias (blinding of participant and personnel), detection bias (blinding of assessor), attrition bias, and other biases (e.g. multiple treatment groups, comparable baseline values, and number of participants).

We used Review Manager (RevMan, version 5.1, The Nordic Cochrane Centre, The Cochrane Collaboration, Copenhagen, Denmark) for meta-analysis. We considered meta-analyses worthwhile only if at least three studies with at least 100 patients per treatment arm were available for analysis. In order to deal with the expected clinical and methodological heterogeneity across studies, a random effects model with inverse variance was applied. For dichotomous data, the Mantel-Haenszel-method was used. Risk ratio and 95% confidence interval (95% CI) were calculated for binary outcome and mean difference (MD) and 95% CI were calculated for continuous outcomes. The Peto odds ratio was used to analyse the risk of respiratory depression, because of the low incidence. The *I*^2^ statistic was used to assess heterogeneity and an *I*^2^>50% was considered important heterogeneity.^16^ A *P*-value of <0.05 was taken to indicate statistical significance. We performed the following prespecified subgroup analyses: laparoscopic surgery, laparotomic surgery, addition of bupivacaine to the intrathecal hydrophilic opioids, solely intrathecal hydrophilic opioids, studies with an ERP, and studies with a sham procedure in the control group for blinding purposes. For the latter, only studies with a lumbar needle insertion in the control group, either s. c. or intrathecally and regardless if medication was administered, were included in this subgroup.

Asymmetry in conventional funnel plots can exist without true asymmetry, and reasons other than publication bias can result in asymmetry.^17^^,^^18^ For this reason, contour-enhanced funnel plots were performed. This was done if there were 10 or more studies in the meta-analyses of the outcomes.^15^ We used the test described by Egger and colleagues ^19^ to test for plot asymmetry.

We hypothesised that the effect of the dose of intrathecal opioid could influence the outcome variables. To test for possible heterogeneity, we performed mixed-effects meta-regression (unrestricted maximum likelihood) to determine the effect of the dose of intrathecal opioid. R version 3.1.3 with the ‘meta’ package (version 4.2–0) and ‘metafor’ package (version 1.9–7) was used.

Furthermore, similar to interim analyses of primary clinical trials, meta-analyses have been found to be prone to Type 1 (falsely positive results) and Type 2 error (falsely negative results) during statistical analysis.^20^^,^^21^. TSA is a method to avoid Type 1 errors and was performed for the primary outcomes of our meta-analyses, in order to consider the risk of random error and better estimate the uncertainty in our findings.^22^^,^^23^ TSA methodology was described elsewhere.^24^ Sequential monitoring boundaries are made to decide whether a trial could be terminated early because of a sufficiently small *P*-value. When the cumulative z-curve crosses the monitoring boundaries, an acceptable small chance of a false-positive result can be assumed. We calculated the required information size allowing for a Type 1 error of 0.05, and Type 2 error of 0.20, with the MD from the effect estimate from the conventional random effects model,^25^ and heterogeneity estimated by the diversity (D2) in the included trials. For the analyses we used TSA Viewer (Version 0.9.5.10 Beta, Copenhagen, Denmark: Copenhagen Trial Unit, Centre for Clinical Intervention Research, Rigshospitalet, 2016).

In order to rate the quality of evidence and strength of recommendation of our primary outcomes, the Grading of Recommendations Assessment, Development, and Evaluation system (GRADE) was used.^26^ We assessed the following criteria: risk of bias, inconsistency, indirectness, imprecision, and publication bias. When one of the earlier-mentioned items was assessed as a risk, the evidence was downgraded by two levels (very serious risk) or one level (serious risk). In addition, when the required information size was not reached or the sequential boundary was not crossed, the evidence was downgraded one level as well. One of the following four grades was assigned: high quality (further research is very unlikely to alter the confidence in the estimate of the effect); moderate quality (further research is likely to alter the confidence in the estimate of the effect); low quality (further research is very likely to alter the confidence in the estimate of the effect); or very low quality (the confidence in the effect estimate is very little).

## Results

**Fig 1:**
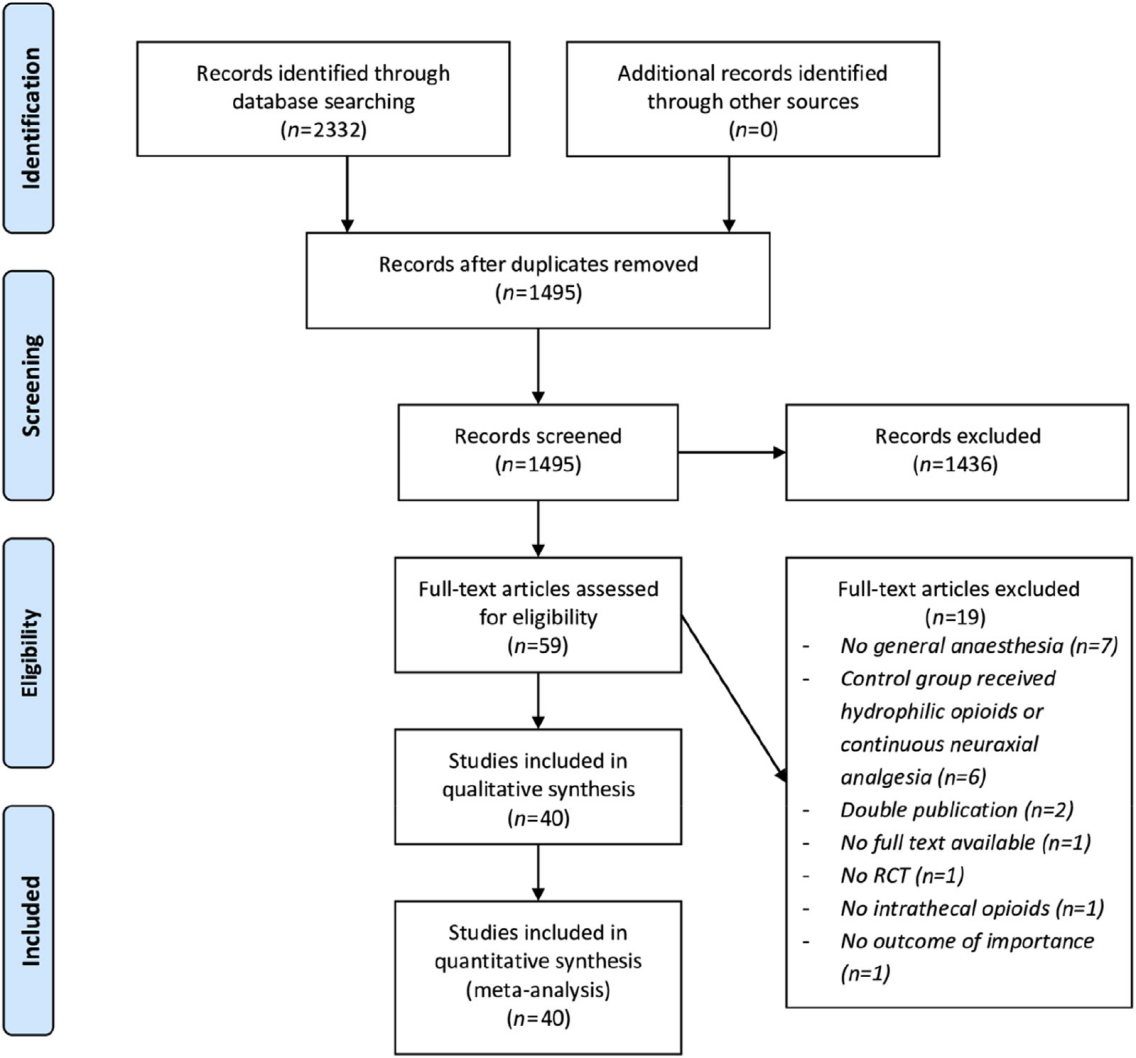
Flow diagram of study selection.

The flow chart of our literature search is presented in Fig 1. A total of 40 studies was included in the quantitative analysis and study characteristics are presented in Table 1. Only Child and Kaufman,^37^ Day and colleagues,^39^ and Levy and colleagues^5^ used diamorphine; all others used morphine as intrathecal opioid. The dose varied between 100 and 800 μg of morphine and except for two studies that administered a body weight adjusted dose of 15 μg kg^−1^ and 50 μg kg^−1^ morphine.^47^^,^^55^.

**Table 1:** Characteristics of included studies. PCA, patient-controlled analgesia; POD, postoperative day.

First author, year of publication, reference	Type of surgery	Number of (intervention *vs* control)	Intervention	Comparator	Postoperative analgesic regimen	Sham procedure	Subgroup	Urinary catheter
**Abd El-Rahman, 2018**^27^	Major abdominal cancer surgery	30 *vs* 30	300 μg morphine, 10 mg bupivacaine, 0.1 mg kg^−1^ ketamine	10 mg bupivacaine, 0.1 mg kg^−1^ ketamine	PCA morphine	Intrathecal medication	A	Unspecified
**Abdel-Ghaffar, 2016**^28^	Major abdominal cancer surgery	30 *vs* 30	500 μg morphine, 10 mg bupivacaine	10 mg bupivacaine	PCA morphine	Intrathecal medication	A	Urinary catheter removed on POD1
**Andreoni, 2002**^29^	Percutaneous nephrolithotomy	9 *vs* 11	0.3–0.5 μg kg^−1^ morphine	Local infiltration with ropivacaine	Unspecified	None	B	Nephrostomy catheter, no urinary catheter
**Andrieu, 2009**^30^	Retropubic radical prostatectomy	17 *vs* 16	4 μg kg^−1^ morphine, maximum of 300 μg	No additional medication	Paracetamol, PCA morphine	None	B, D	Unspecified
**Bae, 2017**^31^	Robotic-assisted laparoscopic prostatectomy	15 *vs* 15	300 μg morphine	No additional medication	PCA morphine, pethidine rescue dose	None	B, C	Urinary catheter for 1 week
**Beaussier, 2006**^32^	Colonic surgery	26 *vs* 26	300 μg morphine	No additional medication	Paracetamol, PCA morphine	S.C. saline	B	Unspecified
**Beltrutti, 2002**^33^	Hysterectomy	15 *vs* 14	4.3 μg kg^−1^ morphine	1.3 μg kg^−1^ buprenorphine i.v.	I.V. buprenorphine	Intrathecal saline	B, D	No postoperative urinary catheter in a part of the patients
**Blay, 2006**^34^	Abdominal aortic surgery	15 *vs* 15	200 μg morphine	No additional medication	Paracetamol, nefopam, morphine rescue dose	S.C. saline	B, D	Urinary catheter of unknown duration
**Boonmak, 2007**^35^	Kidney surgery	40 *vs* 40	300 μg morphine	No additional medication	PCA morphine	None	B, D	Unspecified
**Brown, 2004**^36^	Radical prostatectomy	49 *vs* 50	200 μg morphine, 15 mg bupivacaine, 75 μg clonidine	15 mg bupivacaine, 75 μg clonidine	Paracetamol, ketorolac, PCA morphine	SC saline	A, D	Unspecified
**Child, 1985**^37^	Colonic surgery	8 *vs* 8	50 μg kg^−1^ diamorphine	3–5 μg kg^−1^ fentanyl i.v.	Unspecified	None	B, D	Unspecified
**Colibaseanu, 2019**^38^	Colorectal surgery	98 *vs* 102	100 μg morphine	Bilateral TAP-block with liposomal bupivacaine	Multimodal analgesia, unspecified	None	B, E	Unspecified
**Day, 2015**^39^	Colorectal surgery	60 *vs* 60	250 μg diamorphine, 12.5 mg bupivacaine	10 mg morphine i.v. and PCA morphine	Tramadol and morphine p.o. as needed, diclofenac, paracetamol	None	A, C	Urinary catheter removed on POD1
**Devys, 2003**^40^	Mixed abdominal surgery	30 *vs* 30	300–400 μg morphine	No additional medication	PCA morphine	None	B	Unspecified
**Dichtwald, 2017**^41^	Hepatopancreatic surgery	23 *vs* 26	4 μg kg^−1^ morphine	I.V. loading dose of 0.15 μg kg^−1^ morphine	PCA morphine, paracetamol, and dypirone rescue doses	None	B, D	Urinary catheter of unknown duration
**Downing, 1985**^42^	Cholecystectomy	10 *vs* 10	800 μg morphine	I.V. titration of papaveretum during surgery	I.V. papaveretum rescue dose	None	B, D	Unspecified
**Drasner, 1988**^43^	Major gynaecological surgery	10 *vs* 10	750 μg morphine	I.M. 750 μg morphine	Unspecified	None	B, D	Unspecified
**El-Sherif, 2016**^44^	Laparoscopic bariatric surgery	50 *vs* 50	300 μg morphine, 6 mg bupivacaine	Intrathecal 6 mg bupivacaine and saline	Paracetamol, ketorolac, PCA morphine, wound infiltration with ropivacaine	Intrathecal medication	A, C	Removal of urinary catheter after surgery
**Fléron, 2003**^45^	Abdominal aortic surgery	102 *vs* 115	8 μg kg^−1^ morphine, 1 μg kg^−1^ sufentanil	Continuous i.v. sufentanil	Paracetamol, PCA morphine	None	D	Urinary catheter of unspecified duration
**Hein, 2012**^46^	Abdominal hysterectomy	102 *vs* 34	Mean 200 μg morphine, 12 mg bupivacaine	Intrathecal 12 mg bupivacaine	Paracetamol, PCA morphine	Intrathecal medication	A, D	Unspecified
**Houweling, 1993**^47^	Abdominal aortic surgery	18 *vs* 18	50 μg kg^−1^ morphine	Intrathecal 150 μg sufentanil	500 μg morphine intrathecal	Intrathecal medication	B, D	Urinary catheter of unspecified duration
**Kang, 2019**^48^	Laparoscopic partial hepatectomy	27 *vs* 27	400 μg morphine	Bilateral ESP-block with ropivacaine	Paracetamol, ibuprofen, PCA fentanyl, i.v. meperidine	None	B, C, E	Urinary catheter of unspecified duration
**Kara, 2012**^49^	Major gynaecological surgery	30 *vs* 30	300 μg morphine	No additional medication	PCA morphine	S.C. needle introduction	B	Unspecified
**Karaman, 2006**^50^	Abdominal hysterectomy	12 *vs* 12	5 μg kg^−1^ morphine	No additional medication	Diclofenac, PCA morphine	None	B, D	Unspecified
**Kim, 2016**^51^	Kidney surgery	22 *vs* 23	300 μg morphine	No additional medication	PCA morphine, pethidine rescue dose	None	B, D	Unspecified
**Ko, 2009**^52^	Liver transplantation donors	20 *vs* 20	400 μg morphine	No additional medication	PCA fentanyl	None	B, D	Urinary catheter of unspecified duration
**Kong, 2002**^53^	Laparoscopic colorectal surgery	18 *vs* 17	200 μg morphine, 15 mg bupivacaine	15 mg bupivacaine	PCA morphine	Intrathecal medication	A, C	Unspecified
**Koning, 2018**^3^	Laparoscopic colonic surgery	27 *vs* 29	300 μg morphine, 12.5 mg bupivacaine	I.V. 0.1 mg kg^−1^ piritramide	Paracetamol, diclofenac, PCA piritramide	SC lidocaine	A, C	Urinary catheter removed on POD1
**Koning, 2019**^54^	Robot-assisted radical prostatectomy	76 *vs* 79	300 μg morphine, 12.5 mg bupivacaine	I.V. 0.1 mg kg^−1^ morphine	Paracetamol, diclofenac, PCA morphine	SC lidocaine	A, C	Urinary catheter for one week
**Levy, 2011**^5^	Laparoscopic colorectal surgery	31 *vs* 30	250 μg diamorphine, 12.5 mg bupivacaine	I.V. 10 mg morphine	Paracetamol, diclofenac, tramadol, or morphine	None	A, C	Urinary catheter removed on POD1
**Licina, 1991**^55^	Mixed abdominal surgery	12 *vs* 12	15 μg kg^−1^ morphine	No additional medication	Unspecified	SC saline	B, D	Unspecified
**Marion, 2010**^56^	Abdominal hysterectomy	35 *vs* 32	200 μg morphine, 10 μg fentanyl, 12.5 mg bupivacaine	Intrathecal 10 μg fentanyl, 12.5 mg bupivacaine	Paracetamol, diclofenac, and PCA ketobemidone	Intrathecal medication	A, D	Unspecified
**Motamed, 2000**^57^	Laparoscopic cholecystectomy	17 *vs* 17	100 μg morphine, 5 mg bupivacaine	No additional medication	PCA morphine, paracetamol, and ketoprofen rescue doses	SC saline	A, C	No catheterisation
**Nuri Deniz, 2013**^58^	Retropubic radical prostatectomy	28 *vs* 28	200 μg morphine	No additional medication	PCA tramadol, paracetamol, and diclofenac rescue doses	None	B, D	Unspecified
**Ray, 2017**^59^	Major abdominal surgery	46 *vs* 46	750 μg morphine, 10 mg bupivacaine	I.V. 0.2 mg kg^−1^ morphine, s.c. 0.1 mg kg^−1^ morphine	Paracetamol, SC morphine	Intrathecal saline	A	Urinary catheter of unspecified duration
**Roy, 2006**^60^	Partial hepatic resections	10 *vs* 10	500 μg morphine, 15 μg fentanyl	No additional medication	PCA morphine	S.C. needle introduction	D	Unspecified
**Sarma, 1993**^61^	Abdominal hysterectomy	60 *vs* 20	Mean 300 μg morphine	No additional medication	Pethidine rescue dose	Intrathecal saline	B, D	Urinary catheter removed on POD1
**Selvam, 2018**^62^	Laparoscopic hysterectomy	16 *vs* 15	200 μg morphine, 5 mg bupivacaine	Intrathecal 5 mg bupivacaine	Paracetamol, PCA fentanyl	Intrathecal medication	A, C	Unspecified
**Togal, 2004**^63^	Abdominal hysterectomy	25 *vs* 25	100 μg morphine	No additional medication	PCA morphine	Intrathecal saline	B, D	Urinary catheter removed on POD1
**Wongyingsinn, 2012**^64^	Laparoscopic colonic resection	24 *vs* 25	200 μg morphine, 10 mg bupivacaine	PCA morphine	Paracetamol, naproxen, oxycodone	None	A, C	Urinary catheter removed on POD1

‘No additional medication’ under Comparator means that no additional medication to the postoperative analgesic regimen was administered.

A, addition of bupivacaine to intrathecal hydrophilic opioids; B, only intrathecal hydrophilic opioids; C, laparoscopic procedures; D, open procedures; E, regional anaesthesia.

**Fig 2:**
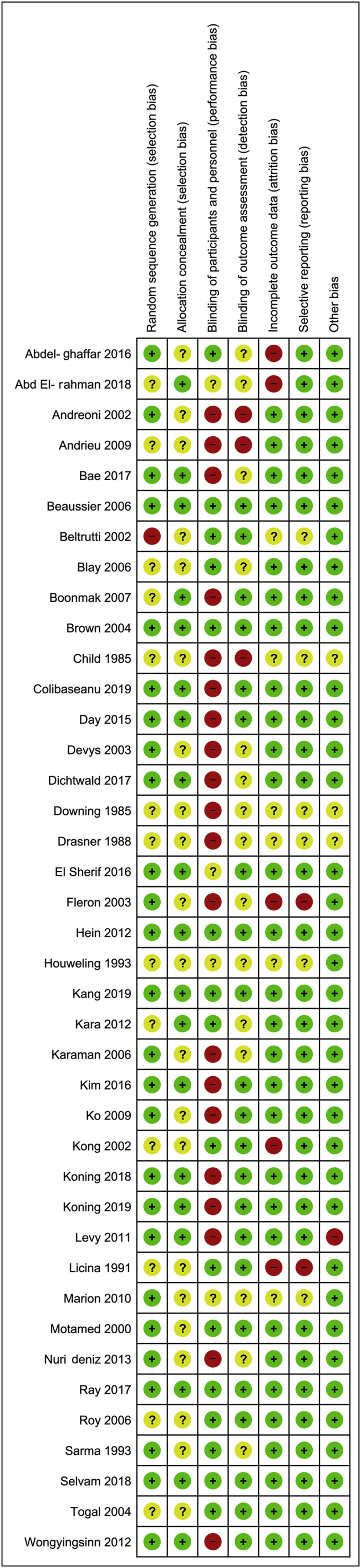
Risk of bias assessment for included studies.

Risk of bias analysis is presented in Fig 2. Main limitations were allocation concealment and blinding of personnel and participants.

### Primary outcomes

**Fig 3:**
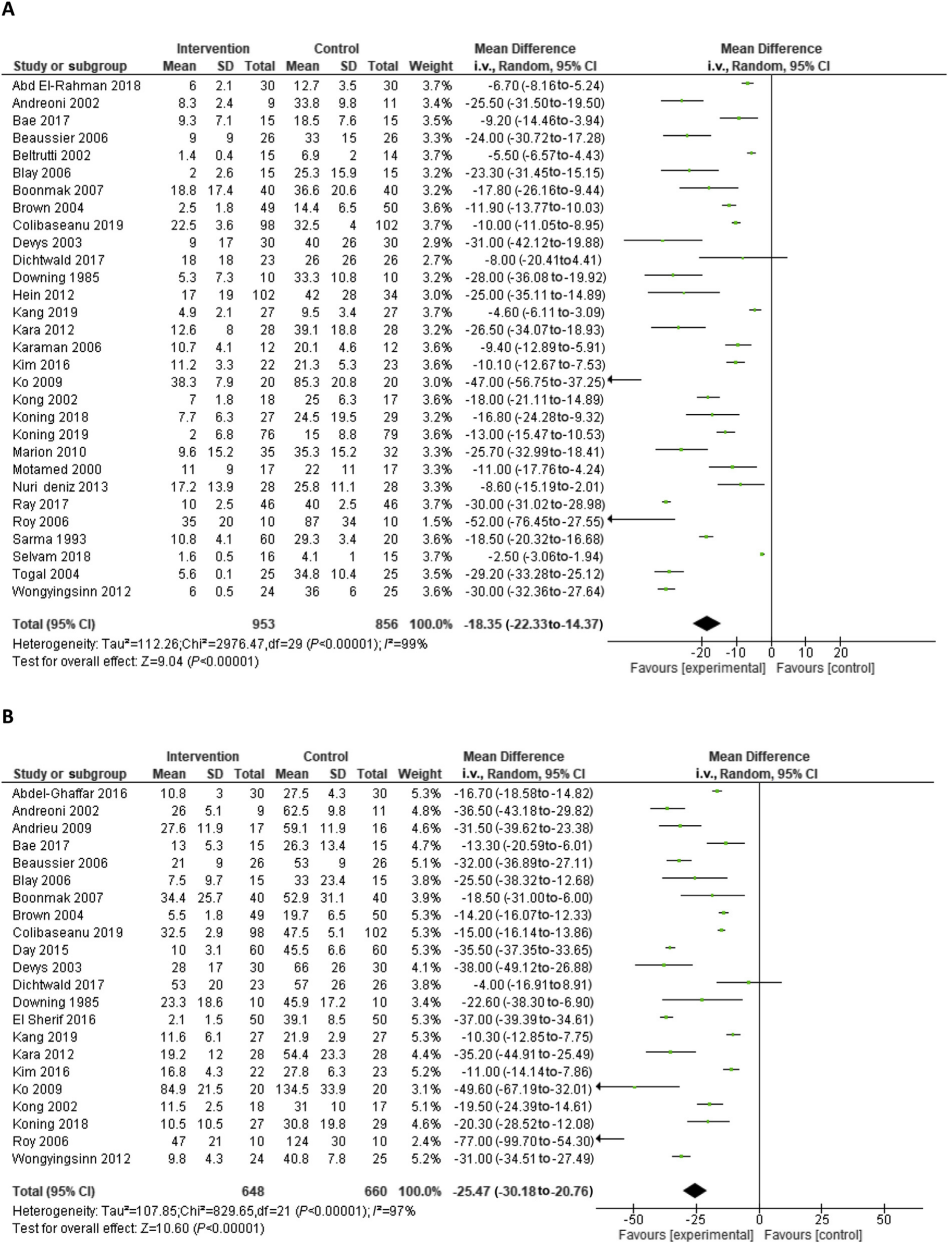
Forest plot of (a) morphine-equivalent consumption after 24 h and (b) 48 h. CI, confidence interval; SD, standard deviation.

Meta-analysis showed an MD in i. v. morphine equivalent consumption after 24 and 48 h of −18.4 mg (95% CI −22.3 to −14.4) and −25.5 mg (95% CI −30.2 to −20.8), respectively, in favour of the intrathecal opioids (Fig 3).

### Secondary outcomes (Table 2)

The pain scores (converted to a range of 0–10) both in rest and during exertion were reduced in the intrathecal opioid group after 24 h. The lower pain scores persisted during exertion after 48 h, but were no longer different in rest. Intraoperative sufentanil-equivalents consumption was reduced, and time-to-first analgesic request was prolonged in the intrathecal opioid group.

**Table 2:** Summary of the meta-analyses. *I*^2^ describes the heterogeneity. RIS, required information size as measured by trial sequential analysis, Egger test describes the risk for publication bias.

Variable	Studies (*n*)	Participants (*n*)	Value (95% CI)	*I*^2^ (%)	RIS	Egger test	Grade
***Benefit***			**Mean difference**				
**Morphine consumption day 1 (mg)**	30	1809	−18.4 (−22.3 to −14.4)	99	266	0.03	High
**Morphine consumption day 2 (mg)**	22	1309	−25.5 (−30.2 to −20.8)	97	103	0.21	High
**Pain scores in rest, day 1 (NRS)**	33	2164	−0.9 (−1.1 to −0.7)	93		0.03	
**Pain in exertion, day 1 (NRS)**	19	1099	−1.2 (−1.6 to −0.8)	79		0.79	
**Pain scores in rest, day 2 (NRS)**	19	1114	−0.4 (−0.7 to −0.1)	97		0.94	
**Pain in exertion, day 2 (NRS)**	13	639	−0.4 (−0.7 to −0.1)	50		0.14	
**Intraoperative sufentanil use (μg)**	11	625	−12.9 (−19.3 to −6.5)	91		0.07	
**Time to first analgesic request (h)**	8	309	9.7 (4.9–14.5)	99		0.01	
**Time to fit-for-discharge (days)**	4	233	−0.3 (−0.5 to −0.1)	28		0.80	
**Length of hospital stay (days)**	17	1416	−0.2 (−0.4 to −0.1)	88		0.12	
***Risk***			**Risk ratio**				
**Incidence of nausea**	25	1412	1.1 (0.9–1.4)	48		0.12	
**Incidence of pruritus**	23	1282	4.3 (2.5–7.5)	57		0.05	
**Incidence of sedation**	12	644	0.7 (0.5–1.1)	2		0.53	
**Incidence of respiratory depression**	31	1862	5.5 (2.1–14.2)	14		0.17	
**Incidence of respiratory depression (<500 μg)**	26	1473	1.1 (0.2–8.2)	21		N/A	

MD, mean difference; 95% CI, 95% confidence interval; NRS, numeric rating scale; RIS, required information size; RR, relative risk.

No increased risk for nausea or sedation was detected. The risk for pruritus was increased. Only Boonmak and colleagues ^35^ reported the incidence of pruritus over different timepoints during the first two postoperative days, thus no data on duration and timing could be retrieved. All other studies reported an incidence of pruritus and monitored over 20–48 h.

Because of the heterogeneity in definition of respiratory depression, only the cases in which medication was administered or mechanical ventilation was necessary were scored as respiratory depression in the meta-analysis. An increased risk for respiratory depression was found between intrathecal and i. v. opioids (Peto odds ratio 5.49 [95% CI: 2.12–14.24]). The incidence of respiratory depression was 18/974 in the intrathecal opioids group *vs* 4/888 in the control group. The timing of respiratory depression after administration of intrathecal opioids was only reported by Dichtwald and colleagues,^41^ which was after a mean of 6 h after injection. Licina and colleagues^55^ and Houweling and Joosten^47^ reported the highest incidence of respiratory depression with 11/12 patients and 2/18 patients, respectively. Both studies also used a much higher dose of intrathecal morphine than the other studies (15 μg kg^−1^ and 50 μg kg^−1^, respectively, resulting in 1200 μg and 4000 μg in a 80 kg patient).

However, when those two outlying high-dose studies were excluded,^47^^,^^55^ the incidence of respiratory depression was 5/944 for the intrathecal opioids group and 4/858 for the control group. This led to a Peto odds ratio of 1.39 (95% CI 0.37–5.21).

The length of hospital stay was reduced with an MD of −0.2 days (95% CI −0.4 to −0.1). In addition, patients in the intervention group were earlier fit-for-discharge as well (−0.3 days [95% CI −0.5 to −0.1]).

Management of urinary catheter was reported in 19 studies (Table 1). The majority inserted a catheter for at least 1 day or for an unspecified duration. These studies reported no interventions for urine retention after removal of the urinary catheter. More specifically, the studies that removed the catheter after 24 h did not report any recatheterisation,^3^^,^^5^^,^^28^^,^^39^^,^^61^^,^^63^ Three studies used no postoperative urinary catheter, which allowed evaluation for urinary retention.^33^^,^^44^^,^^57^ El Sheriff and colleagues^44^ found no urinary retention in 50 patients. Beltrutti and colleagues^33^ found urinary retention in four of seven patients in the intervention group *vs* three of nine patients in the control group, although none required recatheterisation. Motamed and colleagues^57^ found four of 17 patients in the intervention group *vs* one of 17 patients in the control group with urinary retention. Of the four of the intervention group, two were managed with naloxone and two were managed with a urinary catheter.

### Publication bias

The search included trial registries and yielded 26 trial registrations of which 12 were published and already included. Six trials were still recruiting. Two trials were completed and added to the database.^38^^,^^54^ Two other, completed studies were of potential interest but no publication could be found (NCT03620916 and NCT03675646).

**Fig 4:**
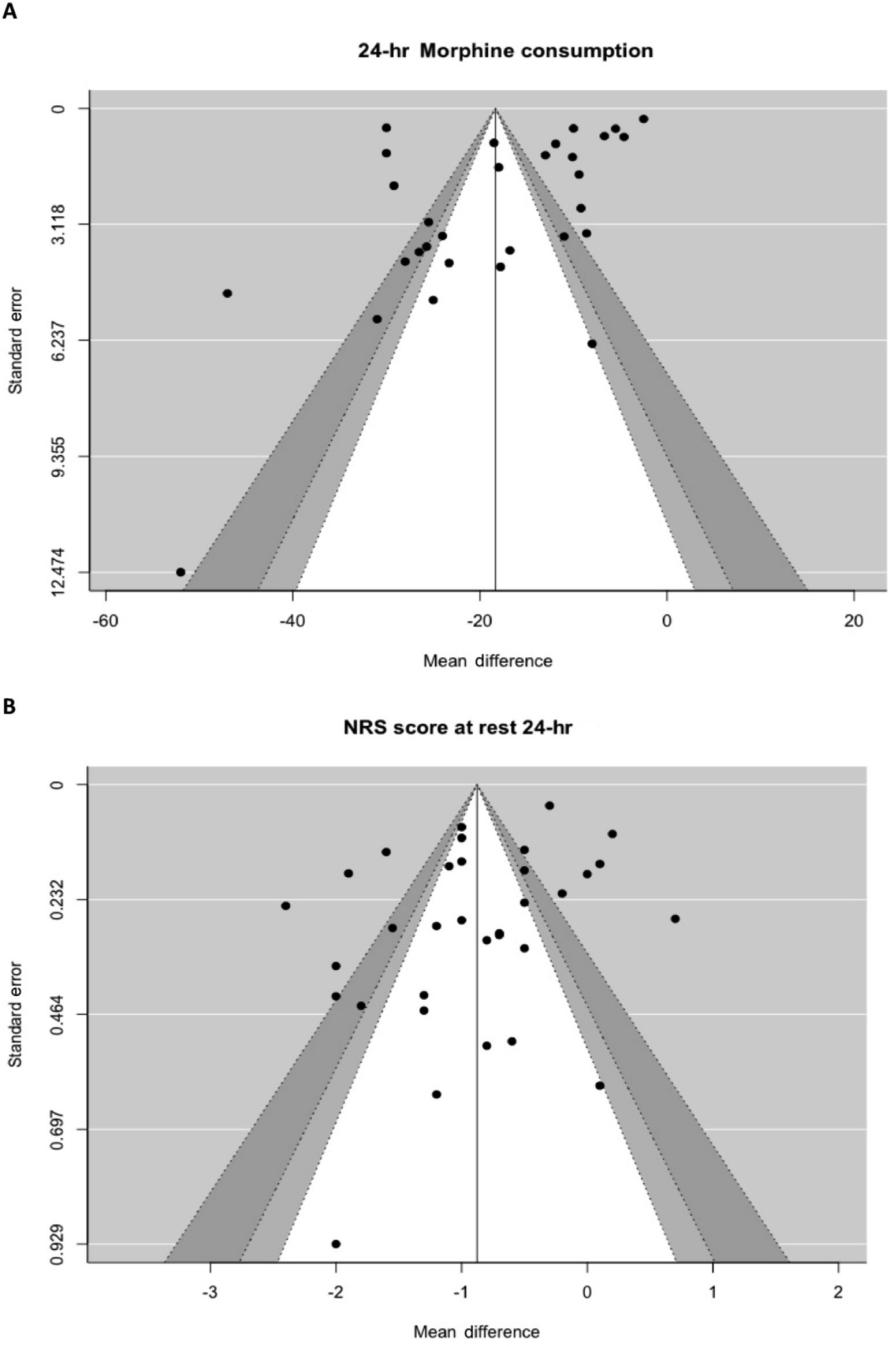
Contour-enhanced funnel plot of A. 24 hour morphine equivalent consumption and B. pain score at rest after 24 hours. NRS, numeric rating scale.

Contour-enhanced funnel plots were generated and only 24 h i. v. morphine equivalent consumption pain scores in rest after 24 h and time to first analgesic request had Egger tests with a *P*-value <0.05 (Fig 4). Asymmetry in the 24 h i. v. morphine equivalents and pain score in rest after 24 h seemed to originate from the lack of studies with low standard error with a large effect size or from the lack of small studies. Based on visual inspection of the two contour-enhanced funnel plots, the asymmetry was unlikely to exaggerate the effect size, which makes a *small study effect* unlikely. The lack of studies with a large benefit and a small standard error is unlikely to be caused by publication bias. Time to first analgesic request included eight studies, which limits its power. The funnel plots are presented in the Supplementary material. Based on these findings, the risk of publication bias seems low.

### Subgroup analyses (see Supplementary material)

Five subgroup analyses were performed, which were solely intrathecal hydrophilic opioids, the addition of intrathecal bupivacaine, laparoscopic surgical procedures, laparotomies, and studies that involved an ERP. The first four mentioned subgroups showed no difference to the general comparison (see Supplementary material). Five studies described use of an ERP.^3^^,^^5^^,^^38^^,^^39^^,^^64^ In these studies the length of stay was −0.2 days (95% CI: −0.5 to 0.1), *I*^2^ 93%. Fit-for-discharge had too few subjects (82 *vs* 84) to produce a reliable analysis. In addition, a sensitivity analysis was performed including only studies with a patient-blinding procedure in the control group for the outcomes ‘pain scores’, morphine consumption, nausea, and pruritus.^3^^,^^27^^,^^28^^,^^32^, ^33^, ^34^^,^^36^^,^^44^^,^^46^^,^^47^^,^^49^^,^^53^, ^54^, ^55^, ^56^, ^57^^,^^59^, ^60^, ^61^, ^62^, ^63^ This analysis showed comparable outcomes to the general comparison.

### Meta-regression

Meta-regression analyses were performed to detect a dose-dependent effect in 24 h and 48 h i. v. morphine equivalents consumption, pain scores in rest and during movement, nausea, pruritus, sedation, and respiratory depression (see Supplementary material). The variation in doses was limited since the most commonly used dose was 300 μg and all but six studies varied between 100 and 400 μg of intrathecal morphine. A dose dependency was observed only for pain scores in rest after 48 h (slope of 0.006/μg morphine [95% CI: 0.001–0.011]) and incidence of pruritus (slope of 0.005/μg morphine [95% CI: 0.002–0.007]) (see Supplementary material).

### Trial sequential analysis

TSA showed a required information size of *n*=266 for 24 h i. v. morphine equivalent consumption, *n*=103 for 48 h i. v. morphine equivalent consumption.

### GRADE recommendations

GRADE recommendations were made for the outcomes ‘i.v. morphine equivalent consumption after 24 h’, ‘i.v. morphine equivalent consumption after 48 h’. Inconsistency was detected, since conventional meta-analyses showed an *I*^2^>74% and a *P*-value for heterogeneity >0.05. The inconsistency was not explained by subgroup analysis or by different types of studies since all studies were prospective randomised trials. Moreover, no studies were in the opposite direction, thus important clinical inconsistency was deemed unlikely. Since the CIs of the outcomes were within a clinical useful range, we did not downgrade the level of evidence because of inconsistency. No publication bias was detected by contour-enhanced funnel plots and all outcomes were directly measured. The risk of bias was high because of limited blinding of participants or outcome assessors in a number of studies, but the sensitivity analysis of only blinded studies with a sham procedure did not show different results. Therefore, insufficient blinding probably had a limited effect and the level of evidence was not downgraded. The required information size was reached for both outcomes. Therefore, we graded the outcomes of 24 and 48 h i. v. morphine equivalent consumption as a high level of evidence.

## Discussion

Our meta-analysis of 40 studies including 2500 patients found a reduced postoperative i. v. morphine equivalent consumption of −18.4 mg (95% CI −22.3 to −14.4) in the first 24 and −25.5 mg (95% CI −30.2 to −20.8) in the first 48 h in the intrathecal hydrophilic opioids group. Moreover, we found clinically relevant reductions by intrathecal hydrophilic opioids for the following secondary outcomes: pain scores in rest and during movement after 24 h, pain scores during movement after 48 h, time to first analgesic request, length of hospital stay, and intraoperative sufentanil equivalent consumption. The risk of pruritus was increased, and a dose-dependent effect was found. Overall, the risk of respiratory depression was increased (Peto odds ratio 5.49 [95% CI: 2.12–14.24]), but when two outlying studies of doses >1000 μg of intrathecal morphine were excluded, a similar incidence of respiratory depression as the control group was found (Peto odds ratio of 1.39 [95% CI 0.37–5.21]). Subgroup analysis for laparoscopic, laparotomic, addition of bupivacaine, and solely hydrophilic intrathecal opioids yielded no substantial differences compared with the total group for all the outcomes.

These results led to different conclusions than the results of a previous meta-analysis.^6^ This meta-analysis shows that the use of intrathecal hydrophilic opioids in abdominal surgery has several benefits including the reduced systemic opioid consumption, lower pain scores, and a slightly reduced length of stay. The risks consist mostly of pruritus. Urinary retention was not evaluated in the majority of the included trials. The risk of respiratory depression was not increased when the studies with a dose more than 1000 μg were excluded. It appeared that a specific indication (i.e. abdominal surgery), a specific definition of respiratory depression, and more recent studies led to an acceptable safety profile. While in the other meta-analysis it was suggested to abandon this analgesic technique, this study shows the positive effects may be substantial in abdominal surgery and the risks are limited.^6^

The reduction in i. v. morphine equivalents consumption may not come as a surprise, since this effect has already been described for many years.^65^ However, we feel that our finding of a reduction in postoperative morphine consumption of 18.4 mg (95% CI −22.3 to −14.4) in the first 24 h is clinically relevant. In addition, difference in morphine consumption further increased to 25.5 mg (95% CI −30.2 to −20.8) after 48 h, a finding that is unique in our study and which was not shown by Meylan and co-workers.^6^ These findings are based on sufficient data, as displayed by TSA.

In addition, the mean morphine equivalent consumption allows a comparison of this method with other opioid-sparing techniques such as i. v. lidocaine (−4.5 mg [95% CI: −6.3 to −2.8]),^66^ high dose pregabalin (−13.4 mg [95% CI: −22.8 to −4.0]),^67^ and ketamine (−10.3 [95% CI −13.8 to −6.8]).^68^ This is not a direct scientific comparison, so it should be interpreted with caution, but it may provide an intuitive effect size. Of importance is that the opioid-sparing effect in our meta-analysis is in addition to paracetamol and NSAIDs, since most studies used this medication as a basal multimodal analgesia regimen. We believe that the use of additional opioid-sparing strategies, such as intrathecal hydrophilic opioids, i. v. lidocaine, pregabalin, or ketamine, should be regarded as addition to the use of paracetamol and NSAIDs, since these are most consolidated in clinical practice.

This work supports the use of intrathecal hydrophilic opioids within an ERP, since the lower pain scores during movement caused by intrathecal hydrophilic opioids may facilitate early mobilisation.^69^ Additionally, other goals such as to minimise systemic opioids and still produce low pain scores are achieved as well.^70^ This mechanism could explain the reduced postoperative length of stay. In line with previous research, we interpreted the difference in length of stay as one out of every five patients leaves the hospital a day earlier, because in most studies the length of stay was scored per full day and not in half or quarter days. Still, this outcome must be interpreted with caution, because the subgroup analysis of studies which implemented an ERP did not show any difference and length of stay may be influenced by non-medical issues, making fit-for-discharge perhaps a better variable for reflecting recovery.^3^

Other studies reported that the use of intrathecal hydrophilic opioids was associated with adverse effects, such as urinary retention, pruritus, nausea, and the risk of late respiratory depression.^71^ By contrast, our meta-analysis was unable to detect a difference in nausea. Urinary retention was not measured since the majority of the included studies used an urinary catheter for at least the first postoperative day. Interestingly, none of these studies reported a case of recatheterisation or urinary retention beyond that period.

The most common side-effect of intrathecal hydrophilic opioids is pruritus and we found a dose-dependent effect for pruritus in the range of 100–800 μg of intrathecal morphine. We have to point out that a previous meta-analysis of Meylan and colleagues^6^ did not detect a dose-dependent effect, which may be attributable to the lower number of studies in that analysis. Studies that have purposely investigated the relationship between the dose and the incidence of pruritus were able to detect a correlation.^72^ Theoretically, severe pruritus might delay hospital discharge, albeit the pruritus probably lasts shorter than the time for recovery. The duration of pruritus was only investigated in the study of Boonmak and colleagues^35^ over 48 h, which showed a decline of incidence after 24 h. This is in accordance with other studies.^3^^,^^73^

Late respiratory depression is an adverse effect of concern and probably limits the widespread use of intrathecal hydrophilic opioids.^74^ Since only one study explicitly investigated the time to respiratory depression, we are unable to draw conclusions on this aspect.^35^ In our analysis we detected similar incidences of respiratory depression (5/944 for the intrathecal opioids group and 4/844 in the control group) by the use of intrathecal opioids in low dosage. This led to a markedly different conclusion than a previous meta-analysis, which found 6/504 in the intrathecal morphine group and 0/440 in the control group. This difference can be explained by a different definition of respiratory depression, the difference in dosage, and the different type of surgery (i.e. abdominal *vs* cardiac surgery).

The definition of respiratory depression varies amongst studies, which makes the incidence and severity of respiratory depression less than clear.^75^ For our analysis, respiratory depression was only scored when a medical intervention (i.e. mechanical ventilation or medication) was installed. This is a high threshold to score respiratory depression, but we believe that this definition excludes respiratory failure as a result of other pathology (e.g. atelectasis, diaphragm dysfunction, pneumothorax, or haemothorax). Meylan and colleagues^6^ used a different definition and included patients after cardiac surgery, who have higher incidences of this type of pathology than abdominal surgery. Although the upside of a high threshold for scoring is that only the clinically important respiratory depression is scored, the downside is the risk of missing respiratory depression that does not require a medical intervention, but still may impact the clinical course of the patients.

Gehling and Tryba^76^ found a dose-dependent effect for respiratory depression with a cut-off of 300 μg. In our meta-regression a dose-dependent effect was visible, but the CI was too wide for statistical significance. In our analysis with the exclusion of two outlying studies, the incidence of respiratory depression that required a medical intervention was still similar to the control group. When excluding these two outlying high-dose studies, the maximum dose included in our analysis was 800 μg, but the majority of the studies used a dose less than 500 μg. For safety measures, we would recommend using doses less than 500 μg, because these doses were predominantly investigated.

The incidence of respiratory depression in our control group seems to be in line with reported incidences in patient-controlled analgesia (PCA) opioids in a Cochrane review.^77^ Still, the Cochrane review used a lower threshold for scoring respiratory depression, making this comparison to be interpreted with caution. However, because the incidences of respiratory depression are likely to be within the same range for low dose intrathecal morphine as for PCA opioids, we suggest that the same monitoring as for patients with PCA opioids should be applied.^77^^,^^78^ The ERAS society recommends this as well.^1^ Nonetheless, coadministration of benzodiazepines and routinely administered systemic opioids should be avoided during the first 24 h, since respiratory depression may occur because of interaction.^79^.

This meta-analysis contains a high level of heterogeneity, which was not explained by the subgroup analysis, meta-regression, or methodological differences of the included studies. The differences in type of surgery is a likely cause of heterogeneity, but further subgroup analysis was not prespecified and could increase the chance of a Type 1 error. The postoperative analgesic regimen consisted in most studies of paracetamol, NSAID, and PCA opioids, but variation adds to heterogeneity as well. Still, the CIs are within clinical significant limits and the effects of individual studies were predominantly in the same direction, therefore we did not alter the GRADE level of evidence based on heterogeneity.

Besides the inherent downside of a meta-analysis by the methodological limitations of the included studies, an additional limitation of this study is the probability of missing studies. We were unable to retrieve a full text of Toǧal and colleagues.^80^ Another issue is the low number of patients for some outcomes. Of importance is the respiratory depression, for which no increased ratio was found. This too could be because of the low number of events and patients. Some outcomes have been reported in dichotomous and continuous variables, such as patient satisfaction and sedation, which limited the ability to pool the data. A third limitation is the pooling of various types of abdominal surgery, which adds to heterogeneity. We mentioned in the introduction that only similar types of surgery should be analysed and even though only abdominal surgery was included, a variance within abdominal surgery is still expected. Subgroup analyses were performed to restrict this limitation. Fourth, not all included studies described characteristics of the recovery phase such as time to oral feeding, mobilisation, and extent of mobilisation and therefore no comments regarding this subject can be made. Finally, high levels of bias for blinding and allocation concealment in the individual studies cause limitations for the meta-analysis as well.

In conclusion, intrathecal hydrophilic opioids reduce intraoperative and postoperative opioid consumption, pain scores, and length of hospital stay in abdominal surgery. These properties make it a potentially important contributor to the overall effects of an ERP, and we feel this technique should be considered more frequently. The risk for pruritus is increased in a dose-dependent fashion. In our opinion, anaesthesiologists are reluctant to administer intrathecal morphine because of fear of respiratory depression. An increased incidence of respiratory depression was found, but this was predominantly caused by two studies using high doses of intrathecal morphine. When these two studies were excluded, this rare complication was not more common in the intervention group than in the control group with systemic opioids. Still, the majority of the studies used a dose less than 500 μg, thus the evidence is predominantly based on this range of doses. We recommend taking similar precautions as with the use of systemically administered opioids for the duration of at least 12 h.

## Authors' contributions

Designed the study: MVK, MK, MAH

Selected the studies: MVK, MK

Extracted the data: MVK, MAH

Performed the analyses: MVK, KR

Interpreted the data: all authors

Drafted the manuscript: MVK

Co-authored the manuscript: MK, KR, RJS, MAH

## Declarations of interest

The authors declare that they have no conflicts of interest.
