# Supplementary material for: Intrathecal hydrophilic opioids for abdominal surgery: a meta-analysis, meta-regression, and trial sequential analysis
Source: Br J Anaesth. 2020 Jul 11;125(3):358–72. doi: 10.1016/j.bja.2020.05.061 (PMC7497029; doi:10.1016/j.bja.2020.05.061)

Supplementary data file

Content:

1. Search terms page 2-4
2. Forest plots page 5-11
3. Funnel plots page 12-18
4. Subgroup analyses page 19-20
5. Meta-regression page 21-25
6. Trial sequential analyses page 26

**Search terms**

**Embase**

('spinal anesthesia'/exp OR 'intrathecal drug administration'/de OR 'intrathecal drug administration':lnk OR 'intradural drug administration'/de OR 'intradural drug administration':lnk OR (rachianaesthes* OR rachianesthes* OR intrathecal* OR intra-thecal* OR intradural* OR subdural* OR ((spinal* OR subarachnoid* OR lumbar*) NEAR/3 (anesthes* OR anaesthes* OR analges* OR block* OR inject* OR techn*))):ab,ti,kw) AND ('morphine derivative'/exp OR 'opiate agonist'/exp OR 'methadone'/de OR 'pethidine'/de OR (morphin* OR morfin* OR diamorphin* OR methadon* OR hydromorphon* OR pethidin* OR meperidin* OR opiate* OR opioid* OR dihydromorphon* OR dilaudid* OR palladone* OR Demerol*):ab,ti,kw) AND ('abdominal surgery'/exp OR 'hysterectomy'/exp OR 'prostatectomy'/exp OR 'kidney surgery'/de OR 'nephrectomy'/exp OR 'nephrotomy'/de OR 'pyelolithotomy'/de OR 'pyeloplasty'/de OR 'pyelotomy'/de OR 'bladder surgery'/exp OR 'adrenalectomy'/de OR 'ovariectomy'/de OR 'salpingooophorectomy'/de OR (laparoscop* OR laparotom* OR laparoendoscop* OR hysterectom* OR uterus-extirpat* OR prostatectom* OR prostat*-resect* OR nephrectom* OR pyelotomy* OR nephrotom* OR pyelolithotom* OR pyeloplast* OR pancreatectom* OR pancreaticoduodenectom* OR pancreaticojejunostom* OR gastrectom* OR gastroduodenostom* OR gastroenterostom* OR gastrojejunostom* OR gastrostom* OR gastrotom* OR cystectom* OR bladder*-reconstruct* OR splenectom* OR adrenalectom* OR cholecystectom* OR ovariectom* OR ovarectom* OR salpingooophorectom* OR salping*-oophorectom* OR salpingoophorectom* OR salpingo-ophorectom* OR adnexectom* OR enterotom* OR ((abdomin* OR abdomen* OR digestiv*-system* OR kidney* OR renal* OR pancrea* OR liver* OR hepat* OR stomach* OR gastric* OR gastro* OR bladder* OR cystic* OR rectum* OR rectal* OR spleen* OR adrenal* OR gallbladder* OR biliar* OR bile-duct* OR ovary OR ovarian OR intestin* OR colon* OR colorect* OR Gynecolog*) NEAR/6 (surger* OR surgical OR operat* OR resect*))):ab,ti,kw) AND ('Controlled clinical trial'/exp OR 'Crossover procedure'/de OR 'Double-blind procedure'/de OR 'Single-blind procedure'/de OR (random* OR factorial* OR crossover* OR (cross NEXT/1 over*) OR placebo* OR ((doubl* OR singl*) NEXT/1 blind*) OR assign* OR allocat* OR volunteer* OR trial OR groups):ab,ti,kw) NOT ([animals]/lim NOT [humans]/lim)

**Cochrane**

((rachianaesthes* OR rachianesthes* OR intrathecal* OR intra-thecal* OR intradural* OR subdural* OR ((spinal* OR subarachnoid* OR lumbar*) NEAR/3 (anesthes* OR anaesthes* OR analges* OR block* OR inject* OR techn*))):ab,ti,kw) AND ((morphin* OR morfin* OR diamorphin* OR methadon* OR hydromorphon* OR pethidin* OR meperidin* OR opiate* OR opioid* OR dihydromorphon* OR dilaudid* OR palladone* OR Demerol*):ab,ti,kw) AND ((laparoscop* OR laparotom* OR laparoendoscop* OR hysterectom* OR uterus-extirpat* OR prostatectom* OR prostat* NEXT resect* OR nephrectom* OR pyelotomy* OR nephrotom* OR pyelolithotom* OR pyeloplast* OR pancreatectom* OR pancreaticoduodenectom* OR pancreaticojejunostom* OR gastrectom* OR gastroduodenostom* OR gastroenterostom* OR gastrojejunostom* OR gastrostom* OR gastrotom* OR cystectom* OR bladder* NEXT reconstruct* OR splenectom* OR adrenalectom* OR cholecystectom* OR ovariectom* OR ovarectom* OR salpingooophorectom* OR salping* NEXT oophorectom* OR salpingoophorectom* OR salpingo-ophorectom* OR adnexectom* OR enterotom* OR ((abdomin* OR abdomen* OR digestiv* NEXT system* OR kidney* OR renal* OR pancrea* OR liver* OR hepat* OR stomach* OR gastric* OR gastro* OR bladder* OR cystic* OR rectum* OR rectal* OR spleen* OR adrenal* OR gallbladder* OR biliar* OR bile-duct* OR ovary OR ovarian OR intestin* OR colon* OR colorect* OR Gynecolog*) NEAR/6 (surger* OR surgical OR operat* OR resect*))):ab,ti,kw)

**Web of Science**

TS=(((rachianaesthes* OR rachianesthes* OR intrathecal* OR intra-thecal* OR intradural* OR subdural* OR ((spinal* OR subarachnoid* OR lumbar*) NEAR/2 (anesthes* OR anaesthes* OR analges* OR block* OR inject* OR techn*)))) AND ((morphin* OR morfin* OR diamorphin* OR methadon* OR hydromorphon* OR pethidin* OR meperidin* OR opiate* OR opioid* OR dihydromorphon* OR dilaudid* OR palladone* OR demerol*)) AND ((laparoscop* OR laparotom* OR laparoendoscop* OR hysterectom* OR uterus-extirpat* OR prostatectom* OR prostat*-resect* OR nephrectom* OR pyelotomy* OR nephrotom* OR pyelolithotom* OR pyeloplast* OR pancreatectom* OR pancreaticoduodenectom* OR pancreaticojejunostom* OR gastrectom* OR gastroduodenostom* OR gastroenterostom* OR gastrojejunostom* OR gastrostom* OR gastrotom* OR cystectom* OR bladder*-reconstruct* OR splenectom* OR adrenalectom* OR cholecystectom* OR ovariectom* OR ovarectom* OR salpingooophorectom* OR salping*-oophorectom* OR salpingoophorectom* OR salpingo-ophorectom* OR adnexectom* OR enterotom* OR ((abdomin* OR abdomen* OR digestiv*-system* OR kidney* OR renal* OR pancrea* OR liver* OR hepat* OR stomach* OR gastric* OR gastro* OR bladder* OR cystic* OR rectum* OR rectal* OR spleen* OR adrenal* OR gallbladder* OR biliar* OR bile-duct* OR ovary OR ovarian OR intestin* OR colon* OR colorect* OR Gynecolog*) NEAR/5 (surger* OR surgical OR operat* OR resect*)))) NOT ((animal* OR rat OR rats OR mouse OR mice OR murine OR dog OR dogs OR canine OR cat OR cats OR feline OR rabbit OR cow OR cows OR bovine OR rodent* OR sheep OR ovine OR pig OR swine OR porcine OR veterinar* OR chick* OR zebrafish* OR baboon* OR nonhuman* OR primate* OR cattle* OR goose OR geese OR duck OR macaque* OR avian* OR bird* OR fish*) NOT (human* OR patient* OR women OR woman OR men OR man)) AND (random* OR factorial* OR crossover* OR cross-over* OR placebo* OR ((doubl* OR singl*) NEAR/1 blind*) OR assign* OR allocat* OR volunteer* OR trial OR groups))

**Medline**

(Anesthesia, Spinal/ OR Injections, Spinal/ OR (rachianaesthes* OR rachianesthes* OR intrathecal* OR intra-thecal* OR intradural* OR subdural* OR ((spinal* OR subarachnoid* OR lumbar*) ADJ3 (anesthes* OR anaesthes* OR analges* OR block* OR inject* OR techn*))).ab,ti,kf.) AND (exp Morphine Derivatives/ OR Analgesics, Opioid/ OR exp Methadone/ OR exp Meperidine/ OR (morphin* OR morfin* OR diamorphin* OR methadon* OR hydromorphon* OR pethidin* OR meperidin* OR opiate* OR opioid* OR dihydromorphon* OR dilaudid* OR palladone* OR Demerol*).ab,ti,kf.) AND (exp Digestive System Surgical Procedures/ OR exp Hysterectomy/ OR exp Prostatectomy/ OR exp Nephrectomy/ OR exp Nephrotomy/ OR Cystectomy/ OR Cystotomy/ OR Cystostomy/ OR Adrenalectomy/ OR exp Ovariectomy/ OR (laparoscop* OR laparotom* OR laparoendoscop* OR hysterectom* OR uterus-extirpat* OR prostatectom* OR prostat*-resect* OR nephrectom* OR pyelotomy* OR nephrotom* OR pyelolithotom* OR pyeloplast* OR pancreatectom* OR pancreaticoduodenectom* OR pancreaticojejunostom* OR gastrectom* OR gastroduodenostom* OR gastroenterostom* OR gastrojejunostom* OR gastrostom* OR gastrotom* OR cystectom* OR bladder*-reconstruct* OR splenectom* OR adrenalectom* OR cholecystectom* OR ovariectom* OR ovarectom* OR salpingooophorectom* OR salping*-oophorectom* OR salpingoophorectom* OR salpingo-ophorectom* OR adnexectom* OR enterotom* OR ((abdomin* OR abdomen* OR digestiv*-system* OR kidney* OR renal* OR pancrea* OR liver* OR hepat* OR stomach* OR gastric* OR gastro* OR bladder* OR cystic* OR rectum* OR rectal* OR spleen* OR adrenal* OR gallbladder* OR biliar* OR bile-duct* OR ovary OR ovarian OR intestin* OR colon* OR colorect* OR gynecolog*) ADJ6 (surger* OR surgical OR operat* OR resect*))).ab,ti,kf.) AND (exp Controlled clinical trial/ OR "Double-Blind Method"/ OR "Single-Blind Method"/ OR "Random Allocation"/ OR (random* OR factorial* OR crossover* OR cross over* OR placebo* OR ((doubl* OR singl*) ADJ blind*) OR assign* OR allocat* OR volunteer* OR trial OR groups).ab,ti,kf.) NOT (exp Animals/ NOT Humans/)

**Google Scholar**

intrathecal|intradural|subdural|"spinal|lumbar anesthesia|anaesthesia|analgesia" morphin|opiate|opioid "abdominal|abdomen|kidney|pancreas|liver|stomach|gastric|cystic|rectum|spleen|ovary|intestinal|colon surgery|surgical|operation|resection" trial

**Forest plots**

1. Pain scores in rest at day 1


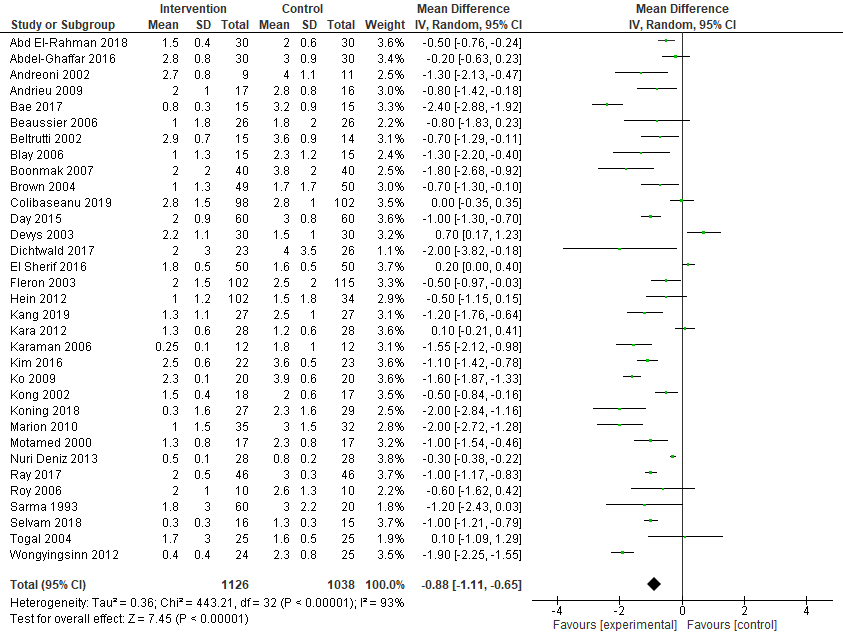


1. Pain scores in exertion at day 1


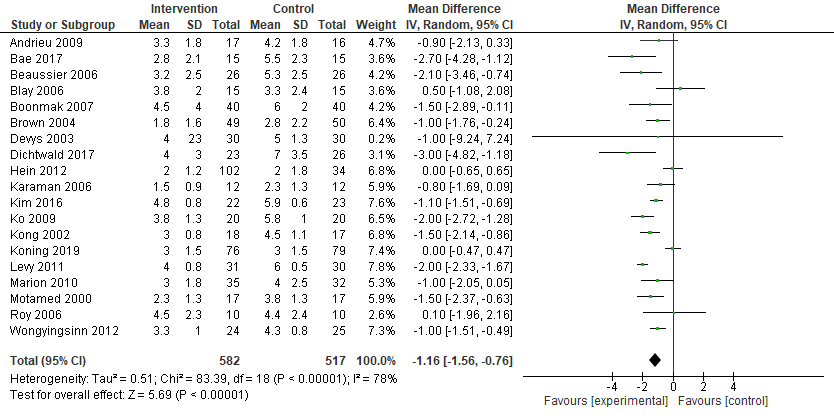


1. Pain scores in rest at day 2


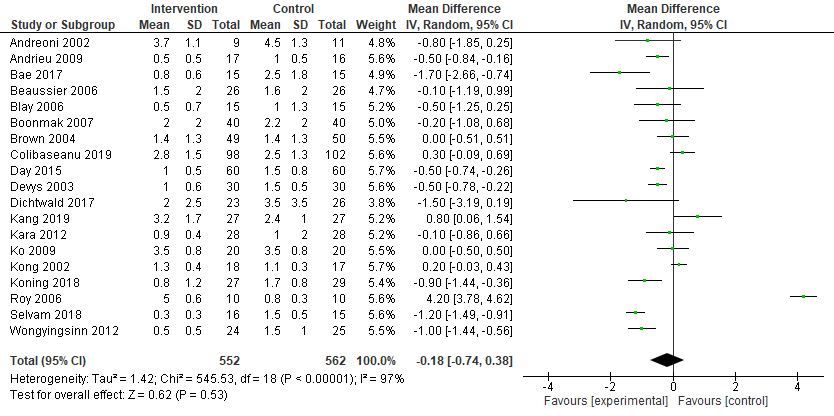


1. Pain scores in exertion at day 2


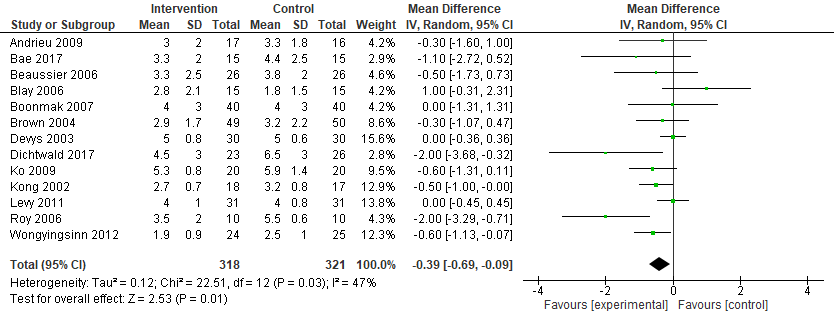


1. Intraoperative sufentanil use


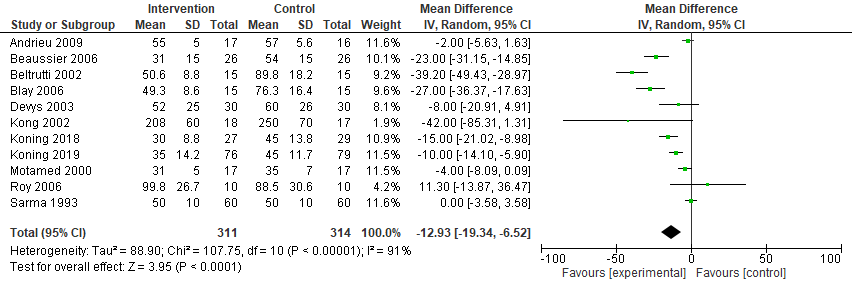


1. Time to first analgesic request. Note that time to first analgesic request is prolonged in the experimental group, which is beneficial thus favours the experimental-group.


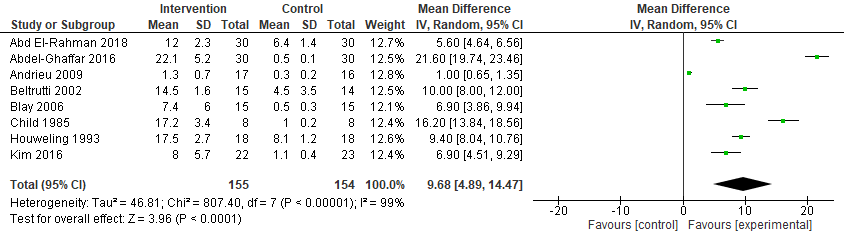


1. Time to Fit for discharge


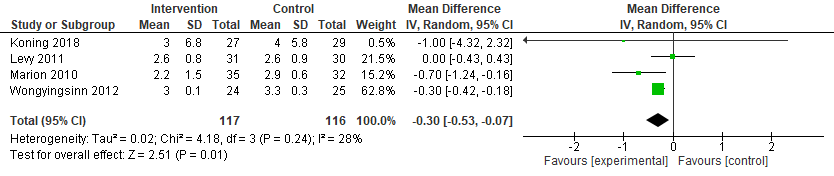


1. Length of hospital stay


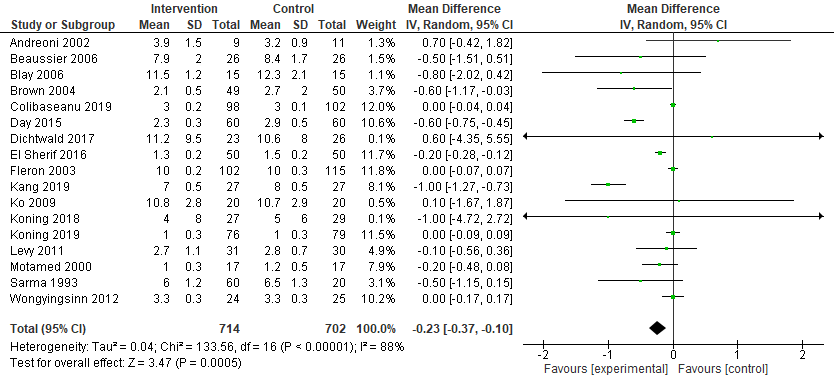


1. Incidence of nausea


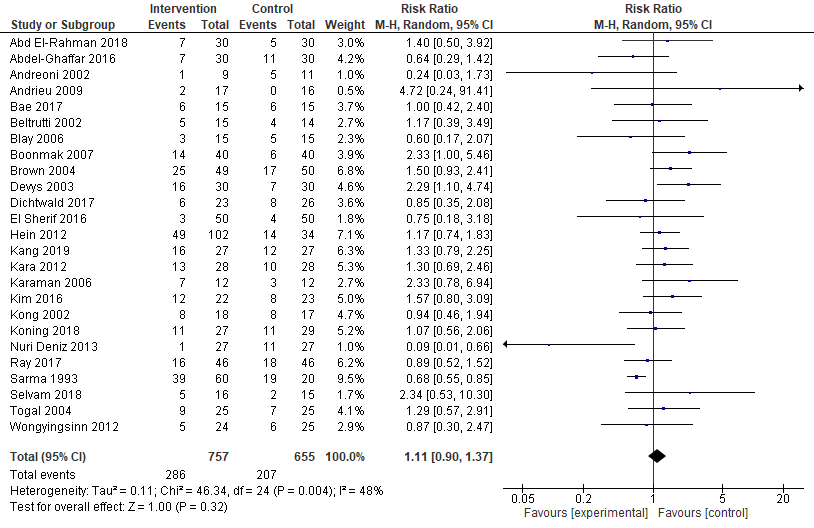


1. Incidence of pruritus. Note that the risk favours the experimental group, which means that the risk for pruritus is higher in the experimental group.


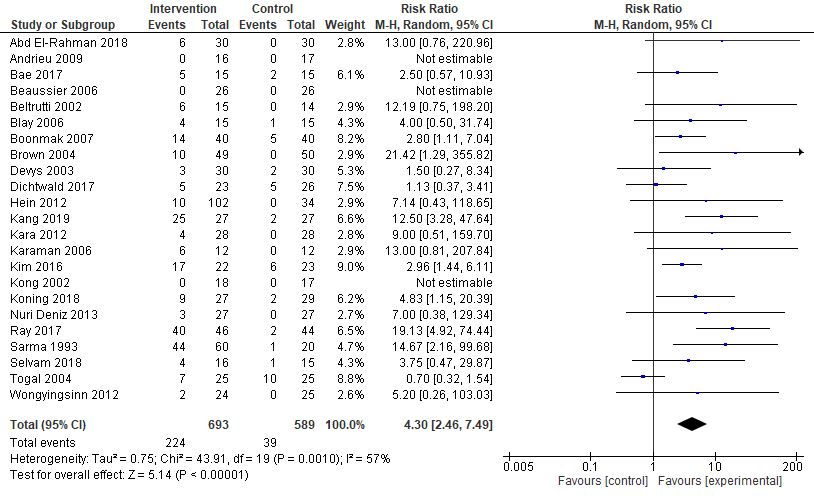


1. Incidence of sedation


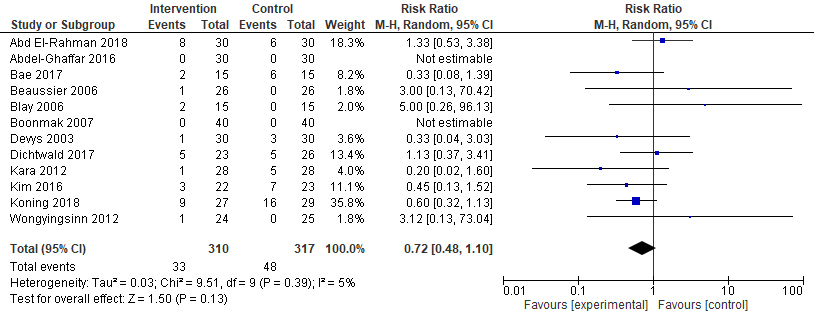


1. Incidence of respiratory depression


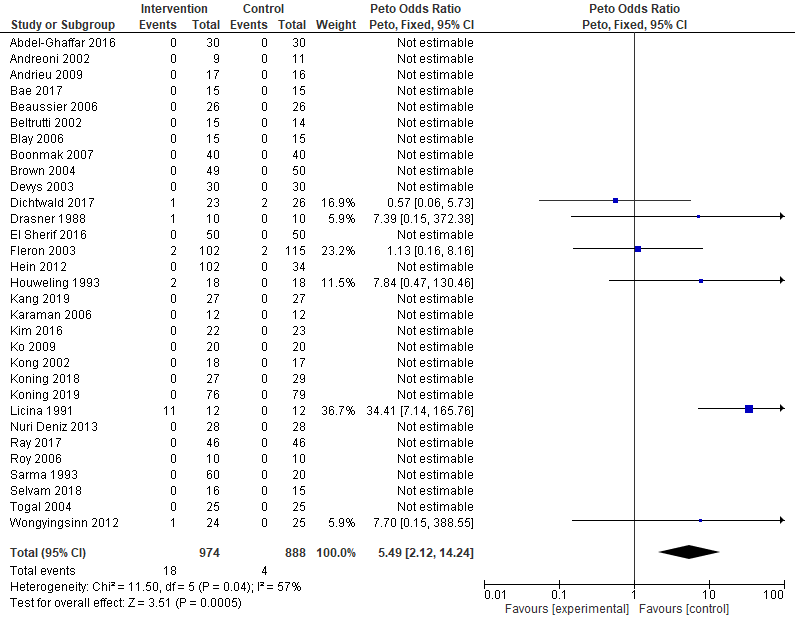


1. Subgroup analysis for calculated mean and standard deviation values for 24 hour morphine equivalent consumption.


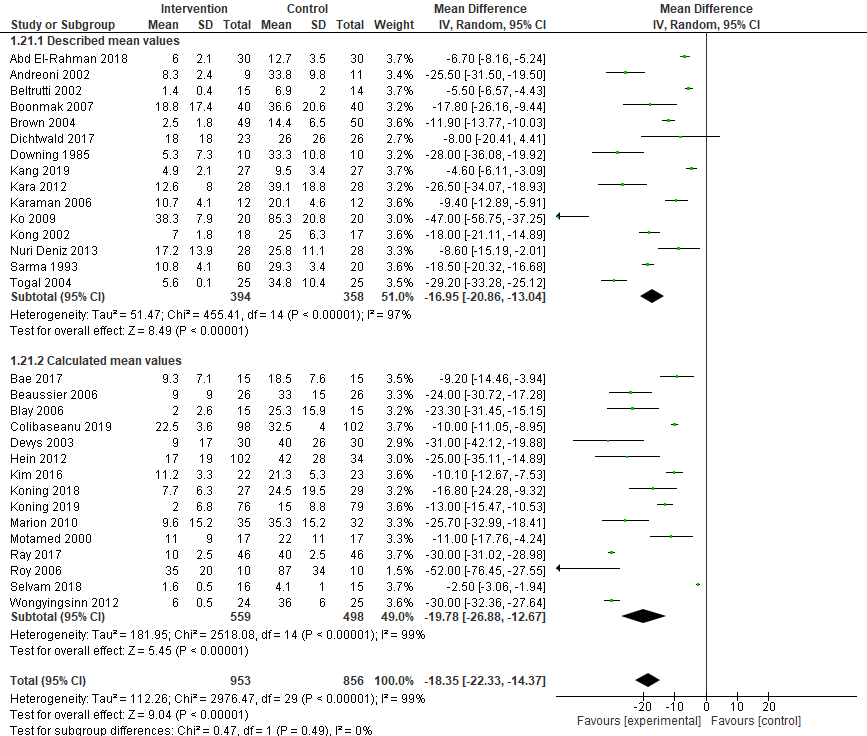


**Funnel plots**

1. Pain scores in rest at day 1


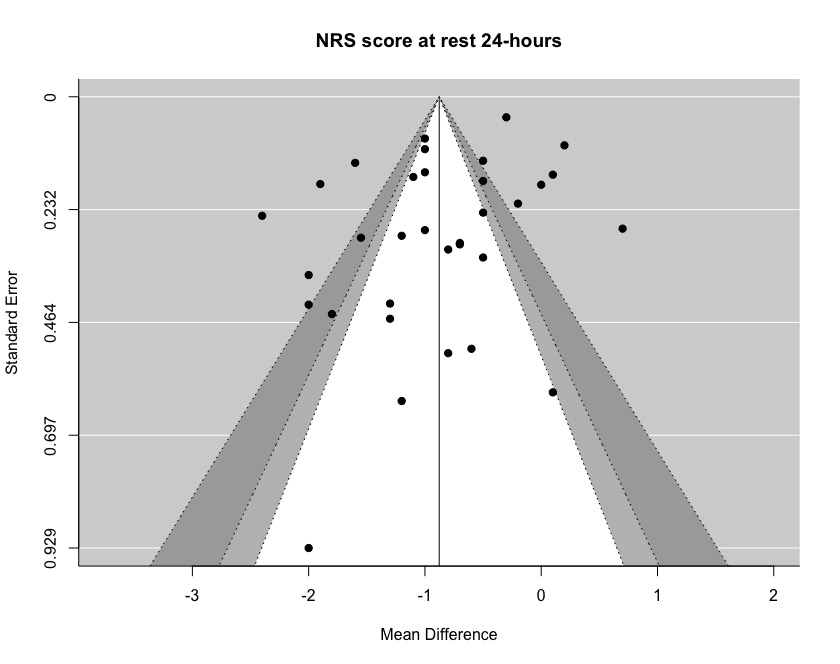


*Egger test p = 0.0264*

1. Pain scores in exertion at day 1


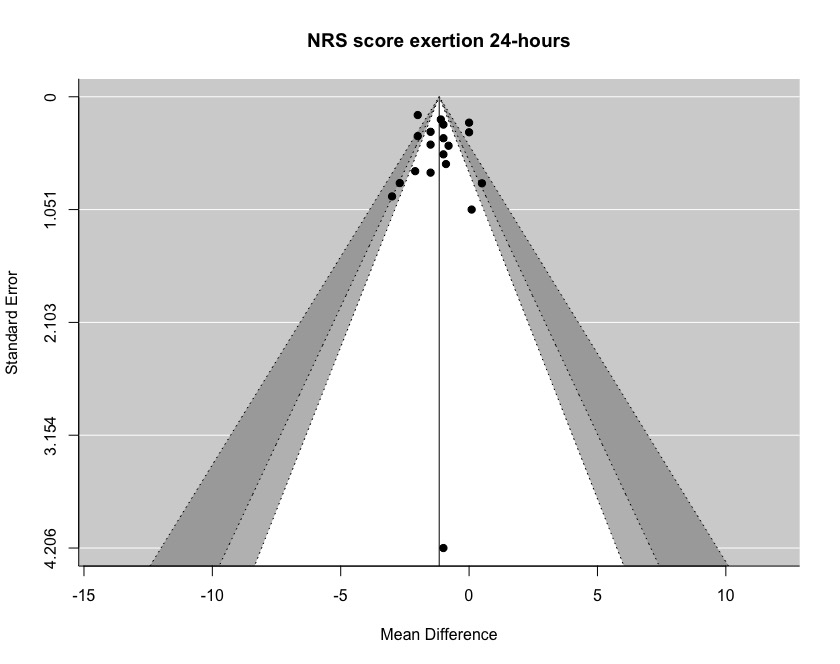


*Egger test p = 0.7928*

1. Pain scores in rest at day 2


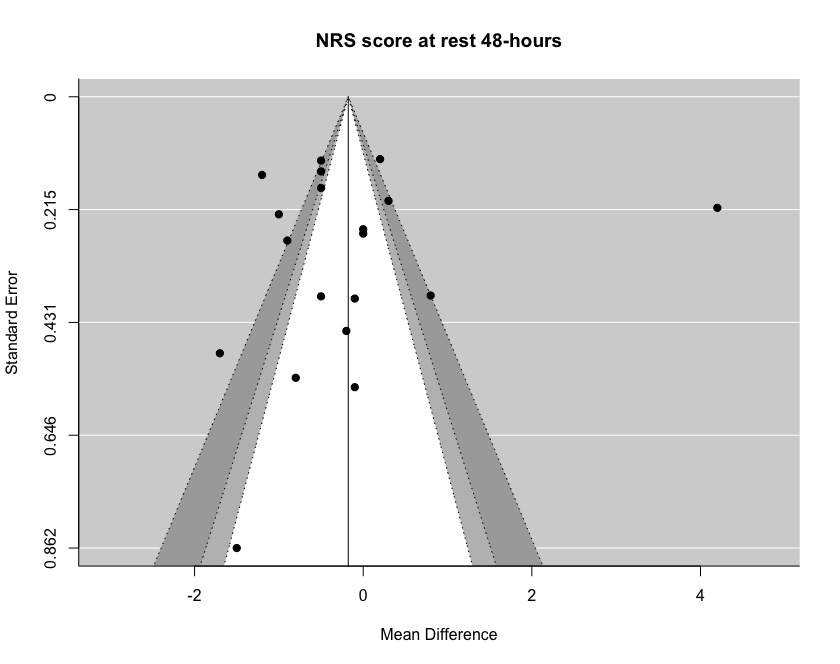


*Egger test p = 0.9445*

1. Pain scores in exertion at day2


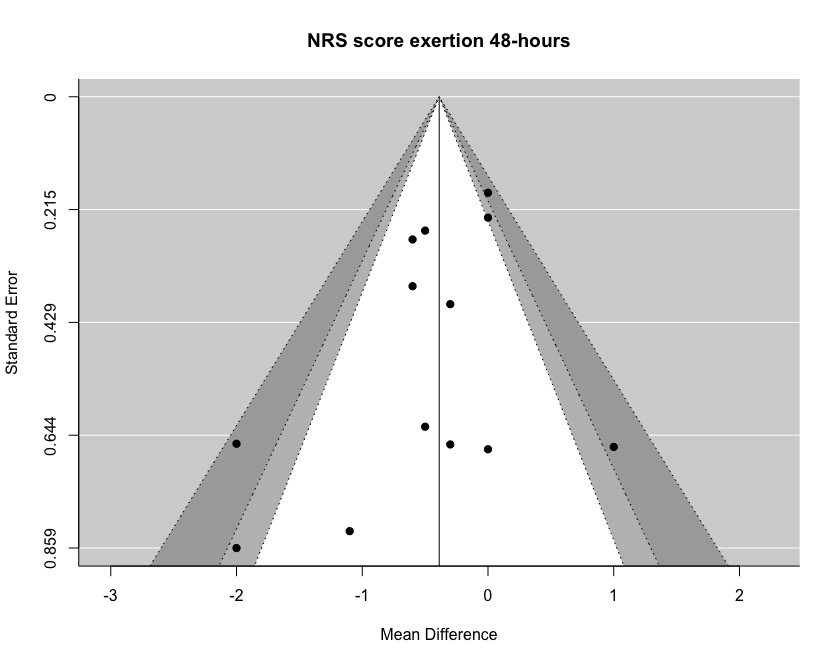


*Egger test p = 0.1418*

1. Morphine consumption at day 1


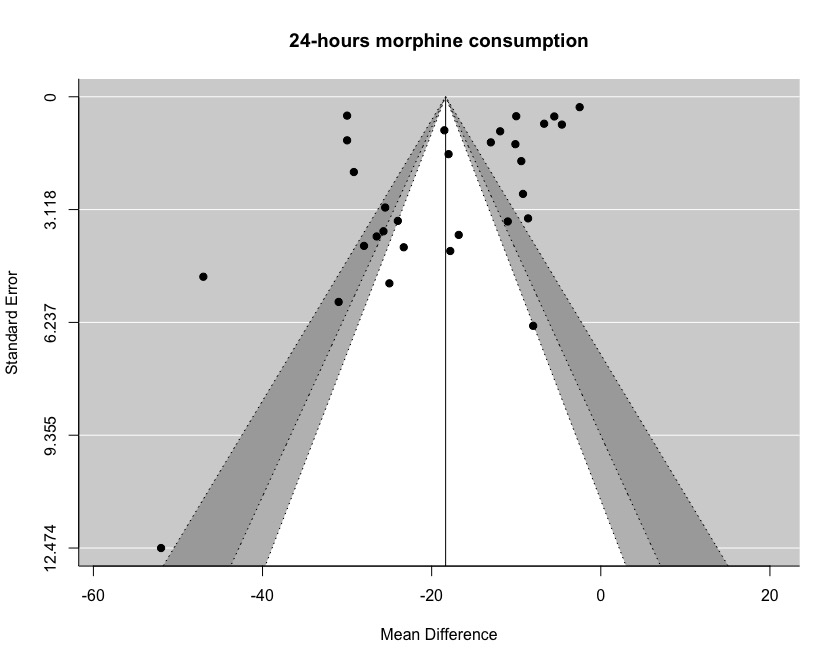


*Egger test p = 0.0321*

1. Morphine consumption at day 2


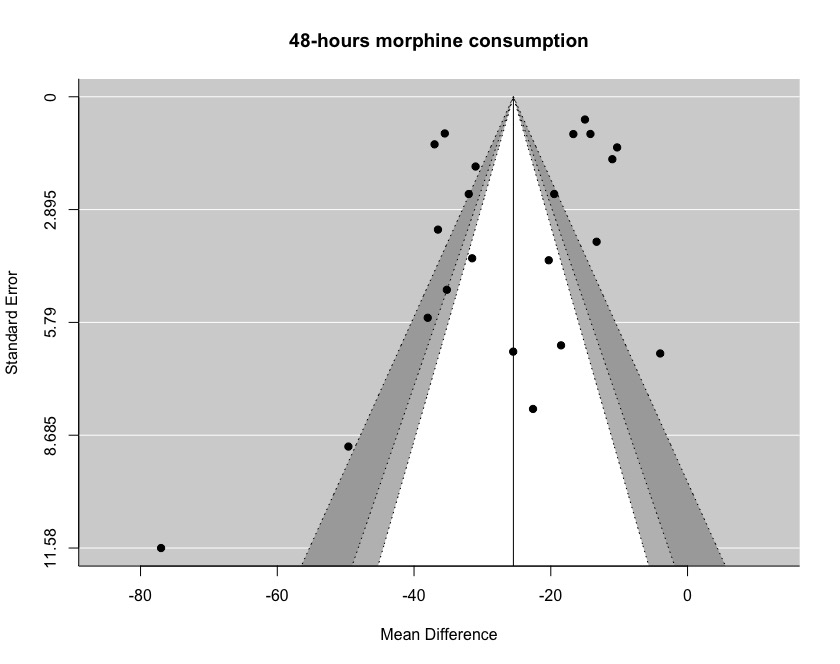


*Egger test p = 0.2111*

1. Intraoperative sufentanil use


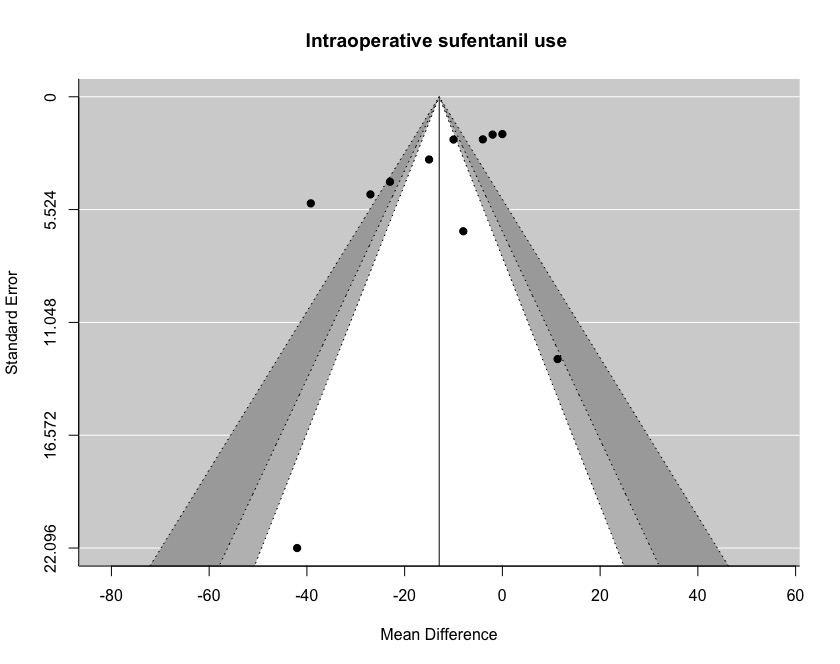


*Egger test p = 0.0682*

1. Time to first analgesic request


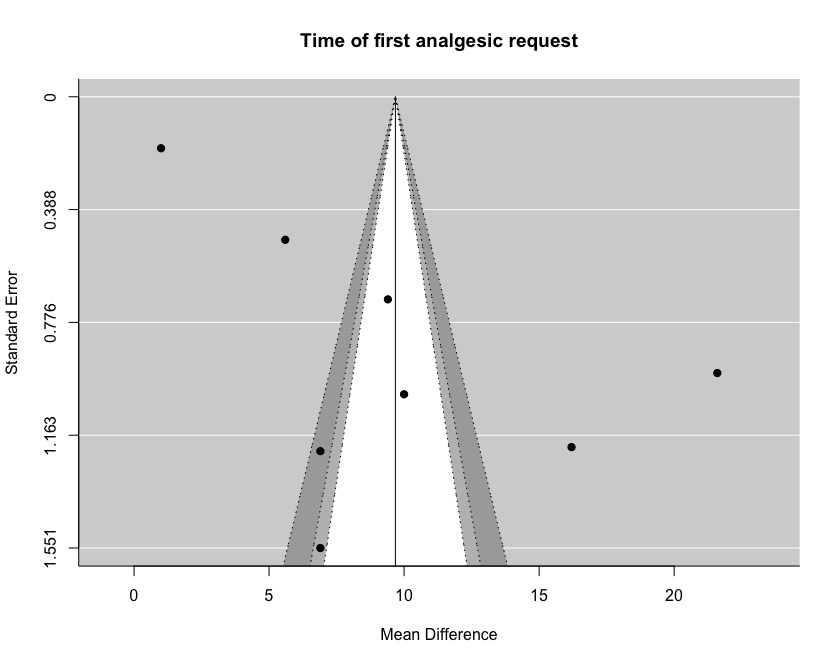


*Egger test p = 0.0085*

1. Time to fit for discharge


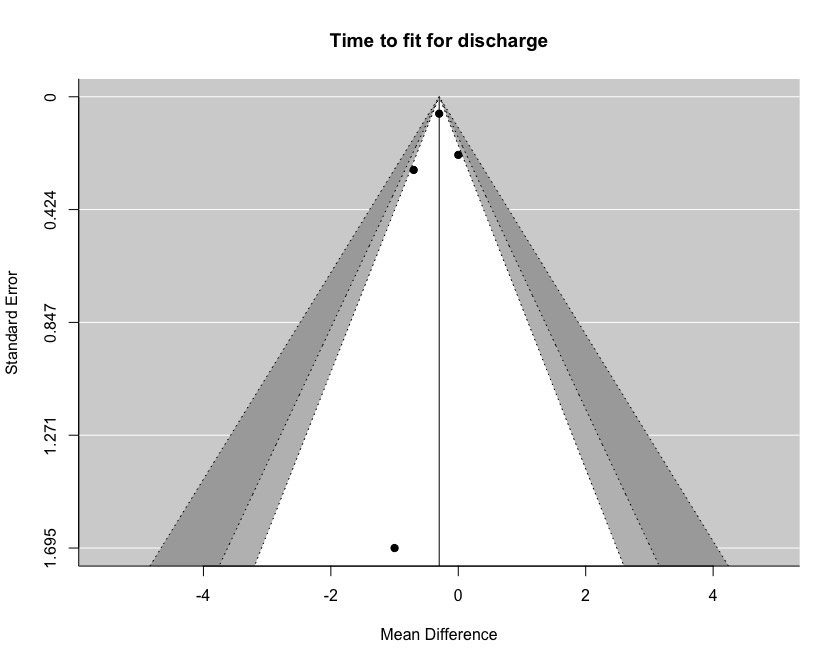


*Egger test p = 0.7996*

1. Length of stay


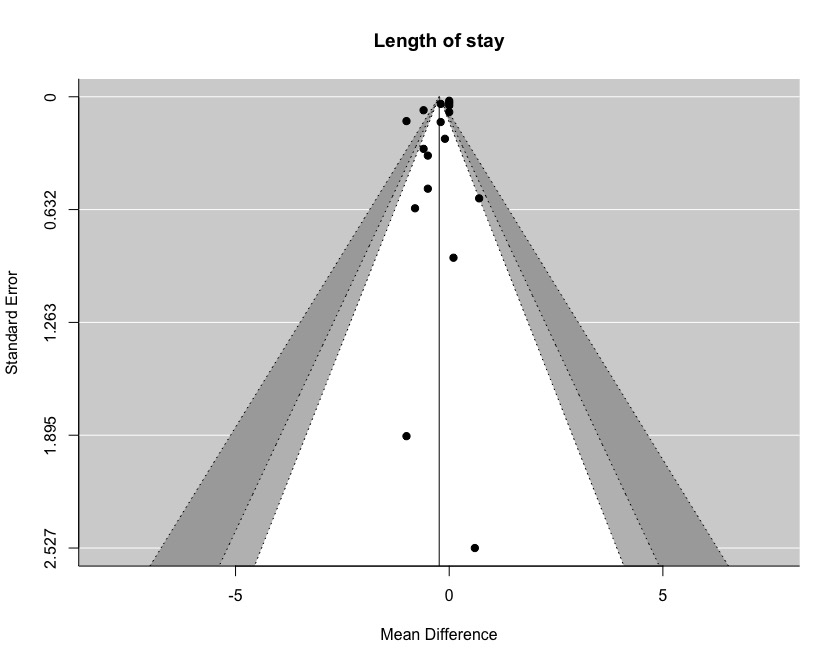


*Egger test p = 0.1160*

1. Incidence of nausea


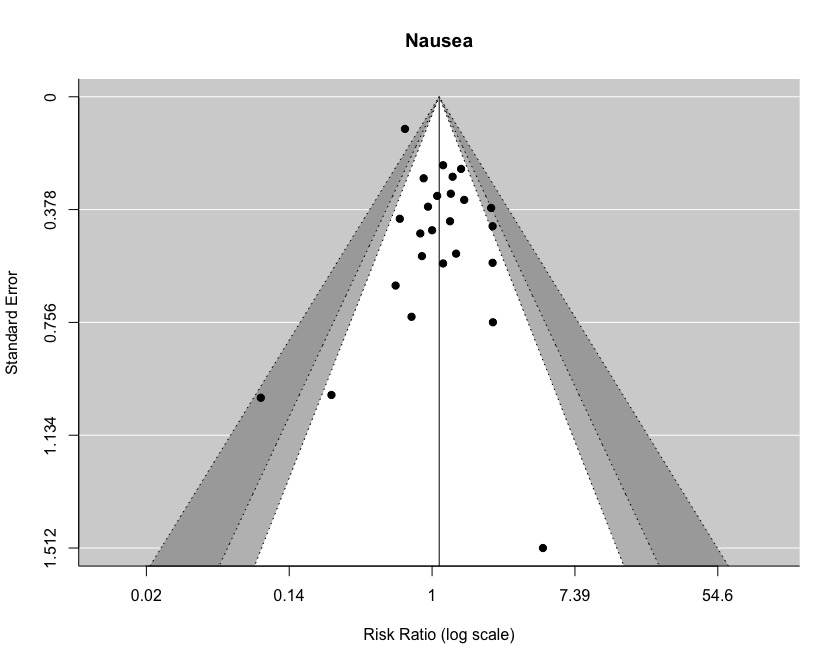


*Egger test p = 0.1196*

1. Incidence of pruritus


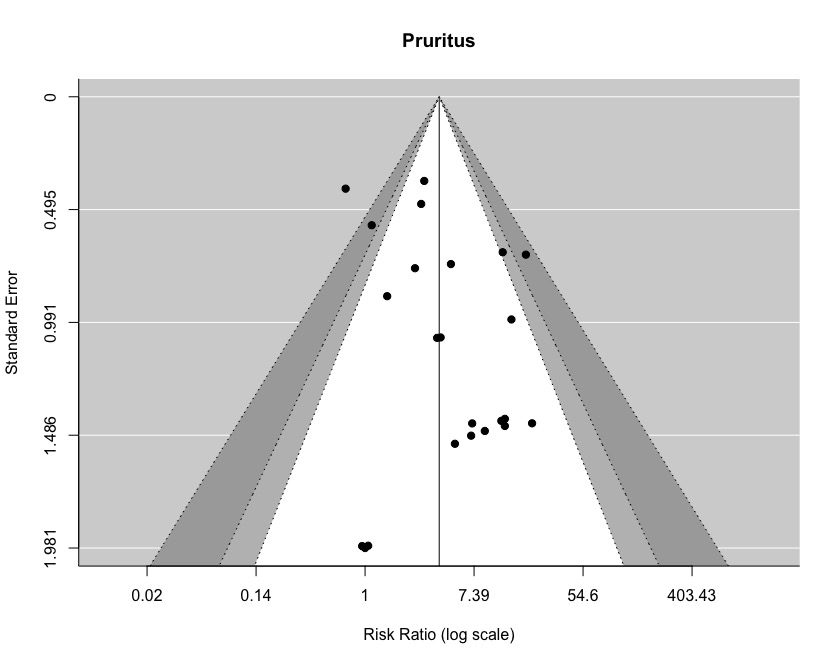


*Egger test p = 0.0509*

1. Incidence of sedation


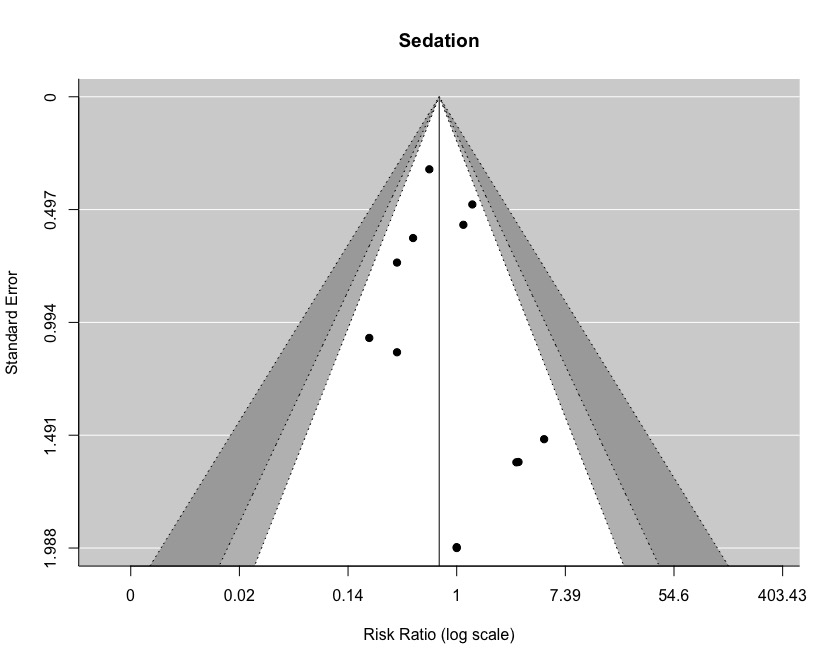


*Egger test p = 0.5255*

1. Incidence of respiratory depression


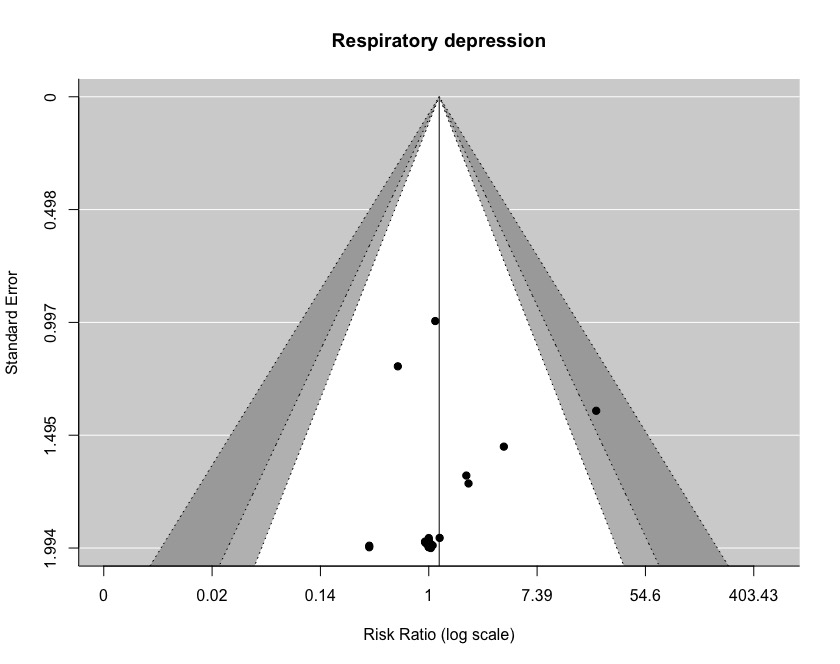


*Egger test p = 0.1724*

**Subgroup analyses**

| Outcome | Number of studies – participants | Effect (mean (95%CI)) | I^2^ |
| --- | --- | --- | --- |
| Pain scores in rest, day 1 (NRS) | 33 – 2164 | -0.9 (-1.1, -0.7) | 93% |
| - *Addition of intrathecal bupivacaine* | 13 – 939 | -0.9 (-1.2, -0.5) | 93% |
| - *Solely intrathecal hydrophilic opioids* | 18 – 988 | -0.9 (-1.3, -0.5) | 93% |
| - *Laparoscopic procedures* | 10 – 529 | -1.1 (-1.7, -0.6) | 95% |
| - *Laparotomic surgery* | 15 – 1035 | -1.0 (-1.4, -0.7) | 91% |
| - *Studies with a placebo or sham procedure* | 18- 1087 | -0.7 (-1.0, -0.4) | 89% |
| Pain in exertion, day 1 (NRS) | 19 – 1099 | -1.2 (-1.6, -0.8) | 79% |
| - *Addition of intrathecal bupivacaine* | 8 – 636 | -1.0 (-1.6, -0.4) | 89% |
| - *Solely intrathecal hydrophilic opioids* | 10 – 443 | -1.4 (-1.9, -0.9) | 56% |
| - *Laparoscopic procedures* | 7 – 384 | -1.3 (-2.0 -0.5) | 89% |
| - *Laparotomic surgery* | 10 – 603 | -1.0 (-1.5, -0.6) | 70% |
| - *Studies with a placebo or sham procedure* | 9 – 628 | -0.8 (-1.3, -0.2) | 73% |
| Pain scores in rest, day 2 (NRS) | 19 – 1114 | -0.4 (-0.7, 0.1) | 97% |
| - *Addition of intrathecal bupivacaine* | 6 – 390 | -0.6 (-1.1, -0.1) | 93% |
| - *Solely intrathecal hydrophilic opioids* | 12 – 704 | -0.3 (-0.6, 0.0) | 68% |
| - *Laparoscopic procedures* | 8 – 395 | -0.5 (-1.0, -0.1) | 91% |
| - *Laparotomic surgery* | 6 – 331 | -0.3 (-0.6, 0.0) | 21% |
| - *Studies with a placebo or sham procedure* | 8 -379 | -0.4 (-0.8, 0.1) | 98% |
| Pain in exertion, day 2 (NRS) | 13 – 639 | -0.4 (-0.7, -0.1) | 50% |
| - *Addition of intrathecal bupivacaine* | 4 – 245 | -0.3 (-0.6, 0.0) | 14% |
| - *Solely intrathecal hydrophilic opioids* | 8 – 374 | -0.3 (-0.8, 0.2) | 47% |
| - *Laparoscopic procedures* | 5 – 196 | -0.6 (-1.1, -0.1) | 61% |
| - *Laparotomic surgery* | 6 – 331 | -0.3 (-0.9, 0.3) | 58% |
| - *Studies with a placebo or sham procedure* | 5 – 236 | -0.5 (-1.1, 0.2) | 62% |
| Morphine consumption day 1 (mg) | 30 -1809 | -18.4 (-22.3, -14.4) | 99% |
| - *Addition of intrathecal bupivacaine* | 11 – 814 | -17.2 (-25.7, -8.8) | 100% |
| - *Solely intrathecal hydrophilic opioids* | 18 – 975 | -17.7 (-21.2, -14.2) | 96% |
| - *Laparoscopic procedures* | 9 – 464 | -15.0 (-22.0, -8.1) | 99% |
| - *Laparotomic surgery* | 14 – 805 | -18.6 (-23.1, -14.0) | 93% |
| - *Studies with a placebo or sham procedure* | 16 – 1022 | -19.7 (-26.1, -13.3) | 99% |
| Morphine consumption day 2 (mg) | 22 – 1308 | -25.5 (-30.2, -20.8) | 97% |
| - *Addition of intrathecal bupivacaine* | 7 – 519 | -25.0 (-33.3, -16.7) | 99% |
| - *Solely intrathecal hydrophilic opioids* | 14 – 769 | -23.4 (-28.4, -18.5) | 93% |
| - *Laparoscopic procedures* | 8 – 464 | -27.9 (-36.9, -18.9) | 98% |
| - *Laparotomic surgery* | 8 – 396 | -19.9 (-25.8 -14.0) | 86% |
| - *Studies with a placebo or sham procedure* | 9 – 508 | -28.0 (-35.6, -20.4) | 97% |
| Time to first analgesic request (hours) | 8 – 309 | 9.7 (4.9, 14.4) | 99% |
| Time to fit-for-discharge (days) | 4 – 233 | -0.3 (-0.5, -0.1) | 28% |
| - *Enhanced Recovery Programs* | 3 – 166 | -0.3 (-0.4, -0.2) | 0% |
| Length of hospital stay (days) | 17 – 1416 | -0.2 (-0.4, -0.1) | 88% |
| - *Addition of intrathecal bupivacaine* | 8 – 674 | -0.2 (-0.4, 0.0) | 87% |
| - *Solely intrathecal hydrophilic opioids* | 8 – 525 | -0.3 (-0.9, 0.2) | 88% |
| - *Laparoscopic procedures* | 8 – 629 | -0.3 (-0.5, -0.1) | 92% |
| - *Laparotomic surgery* | 6 – 515 | -0.3 (-0.6, 0.1) | 38% |
| - *Enhanced Recovery Programs* | 5 – 486 | -0.2 (-0.5, 0.1) | 93% |
| Intraoperative sufentanil use (mcg) | 11—625 | -12.9 (-19.3, -6.5) | 91% |
| - *Addition of intrathecal bupivacaine* | 4 – 280 | -10.0 (-16.2, -3.8) | 75% |
| - *Solely intrathecal hydrophilic opioids* | 6 – 325 | -16.1 (-27.0, -5.1) | 95% |
| Incidence of nausea | 25 – 1412 | 1.1 (0.9, 1.4) | 48% |
| - *Addition of intrathecal bupivacaine* | 10 – 718 | 1.1 (0.9, 1.4) | 0% |
| - *Solely intrathecal hydrophilic opioids* | 15 – 694 | 1.2 (0.8, 1.6) | 65% |
| - *Laparoscopic procedures* | 7 -355 | 1.1 (0.8, 1.5) | 0% |
| - *Laparotomic surgery* | 12 – 709 | 1.1 (0.8, 1.7) | 68% |
| - *Studies with a placebo or sham procedure* | 14 – 914 | 1.0 (0.8, 1.3) | 42% |
| Incidence of pruritus | 23 -1282 | 4.3 (2.5, 7.5) | 57% |
| - *Addition of intrathecal bupivacaine* | 8 – 556 | 8.9 (4.2, 18.9) | 0% |
| - *Solely intrathecal hydrophilic opioids* | 15 – 726 | 3.2 (1.7, 6.0) | 59% |
| - *Laparoscopic procedures* | 6 -255 | 5.3 (2.6, 11.1) | 0% |
| - *Laparotomic surgery* | 12 – 709 | 3.4 (1.6, 7.0) | 66% |
| - *Studies with a placebo or sham procedure* | 13 – 804 | 6.4 (2.3, 18.1) | 70% |
| Incidence of sedation | 12 – 644 | 0.7 (0.5, 1.1) | 2% |
| Incidence of respiratory depression | 31 – 1862 | 2.4 (0.8, 7.4) | 14% |
| - *Addition of intrathecal bupivacaine* | 10 – 813 | 7.7 (0.2, 388.6) | N/A |
| - *Solely intrathecal hydrophilic opioids* | 19 – 812 | 9.0 (2.9, 27.8) | 64% |
| - *Laparoscopic procedures* | 9 – 530 | 3.3 (0.1, 83.9) | N/A |
| - *Laparotomic surgery* | 17 – 1048 | 3.7 (0.6, 24.5) | 57% |
| Mortality | 6 – 554 | 0.4 (0.1, 1.1) | 0% |

*Data presented in mean difference (95% CI) for continuous outcomes and RR (95% CI) for dichotomous variables (i.e. incidences). For the incidence of respiratory depression the Peto Odds Ratio was calculated.*

**Meta-regression**

1. Pain scores in rest at day 1


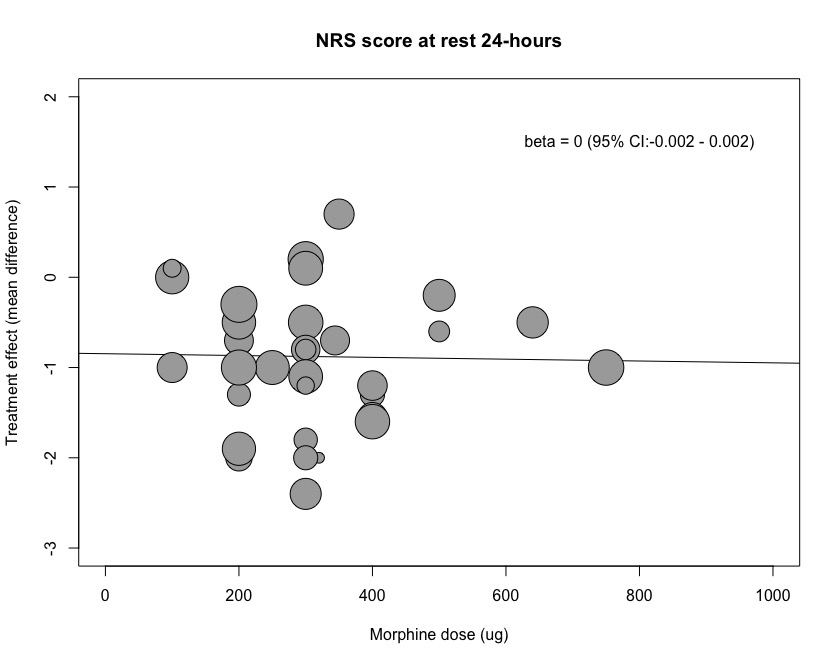


1. Pain scores in exertion at day 1


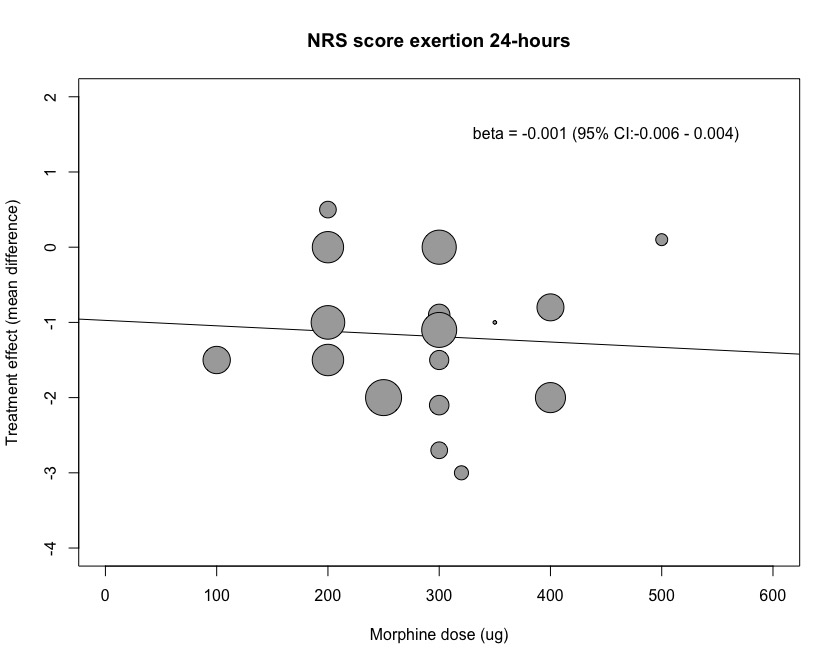


1. Pain scores in rest at day 2


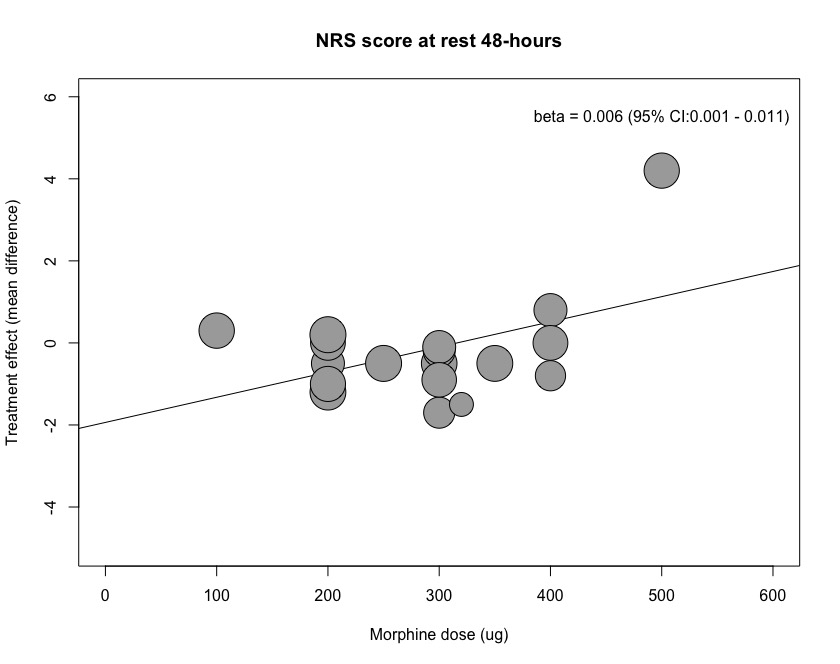


1. Pain scores in exertion at day 2


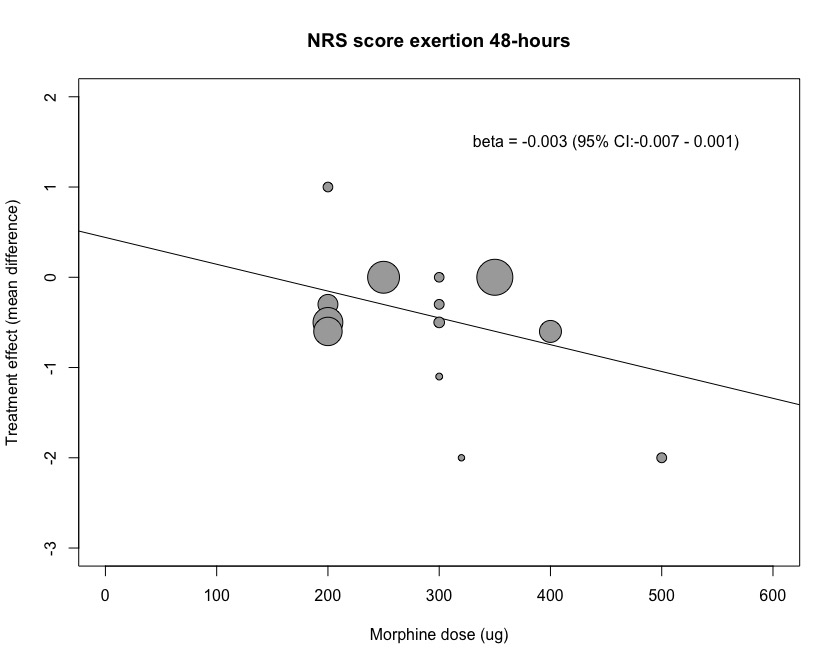


1. Morphine consumption at day 1


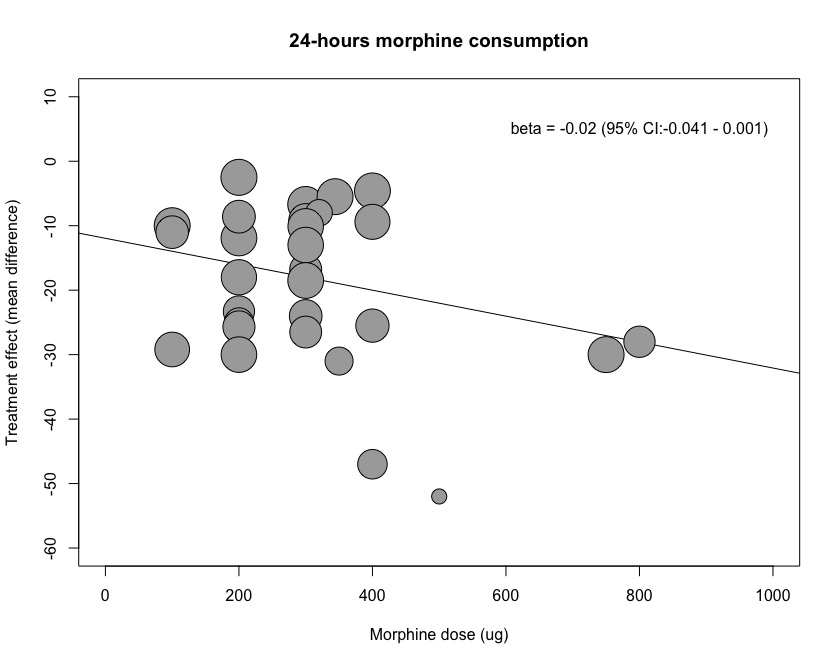


1. Morphine consumption at day 2


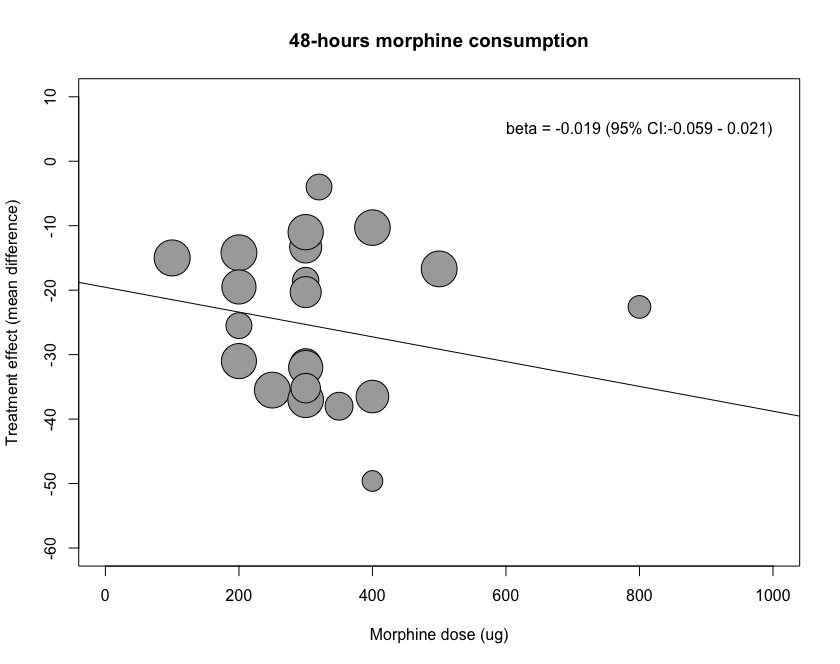


1. Incidence of nausea


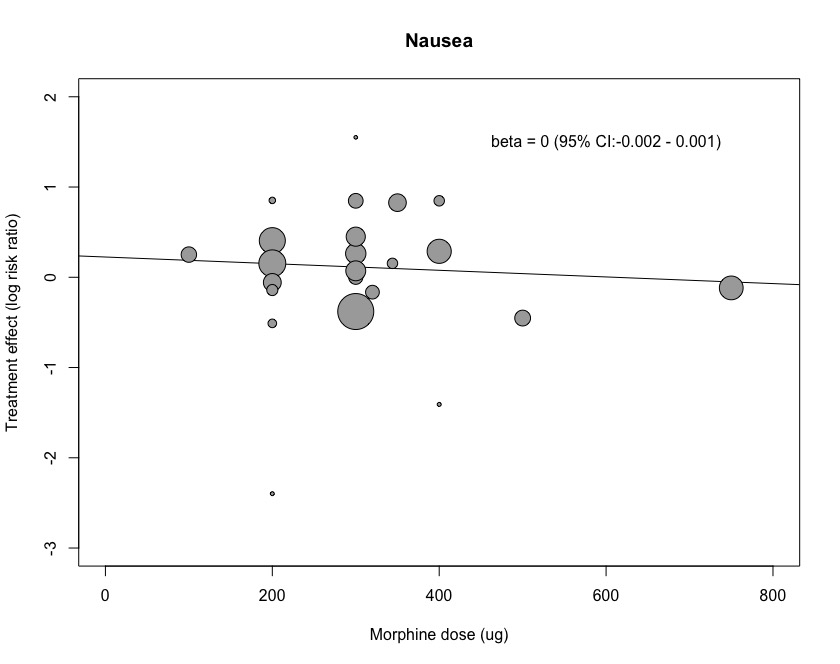


1. Incidence of pruritus


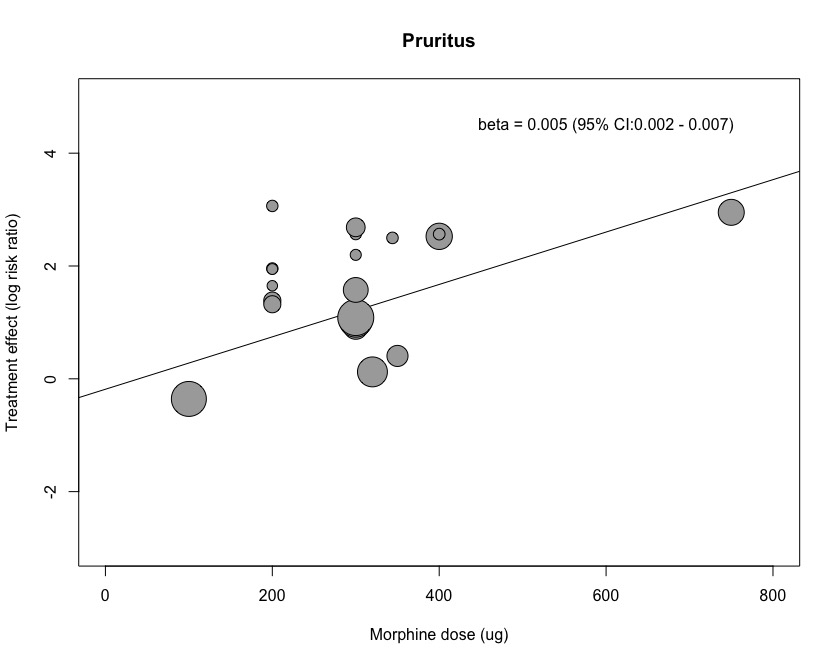


1. Incidence of sedation


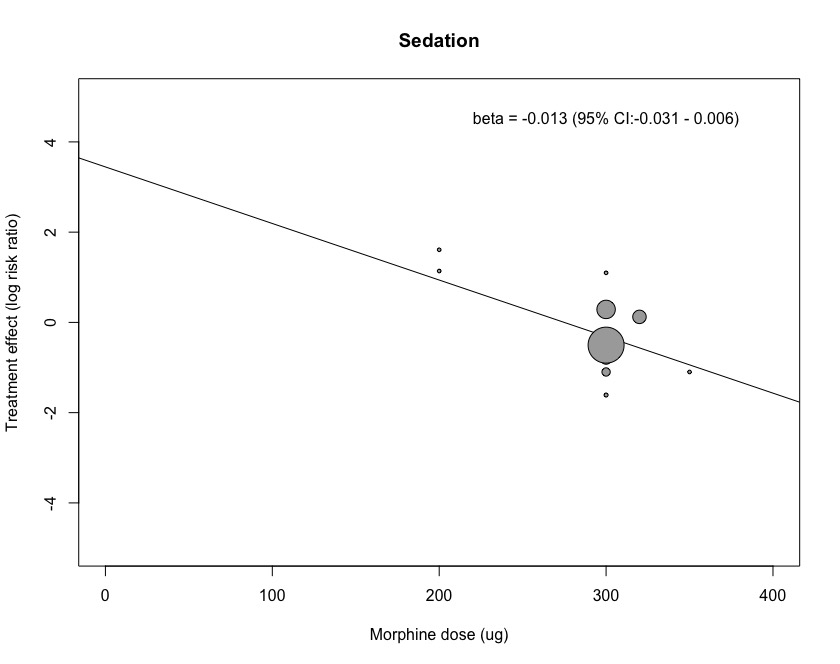


1. Incidence of respiratory depression


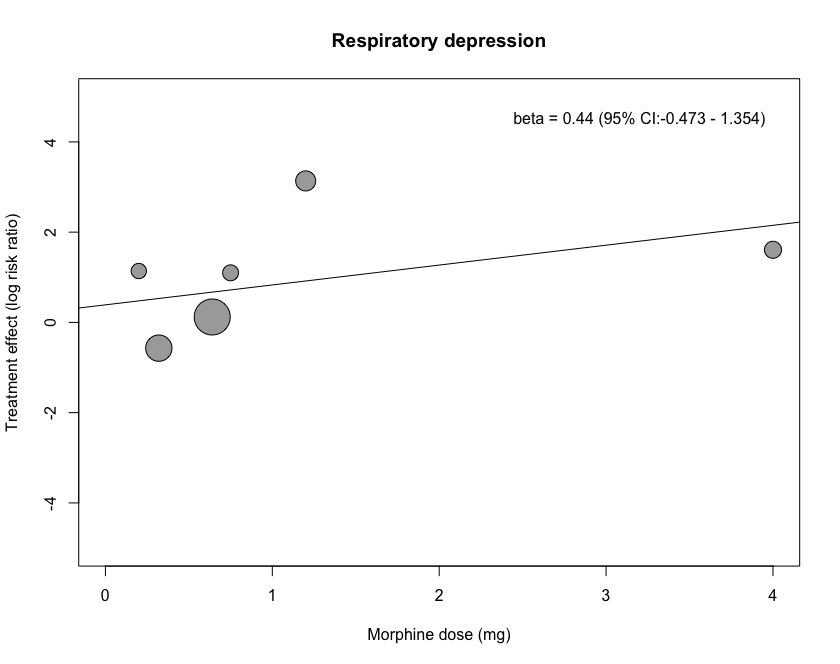


**Trial Sequential Analyses**

1. Morphine consumption at day 1


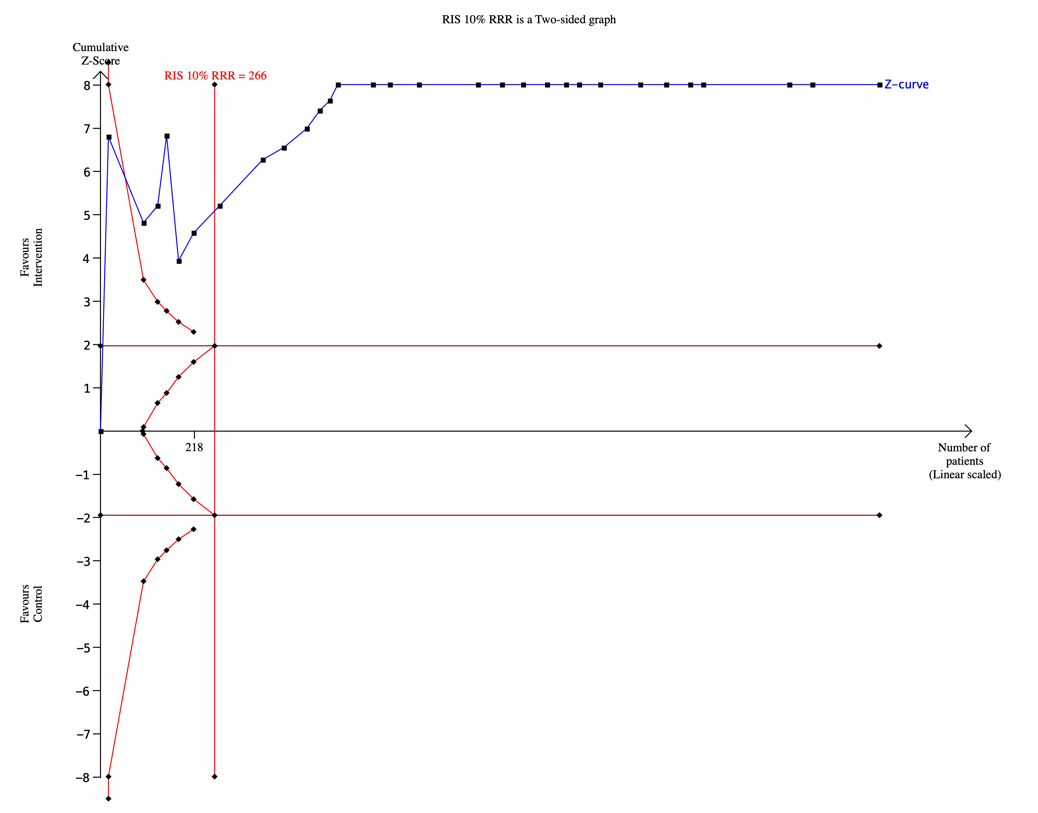


1. Morphine consumption at day 2


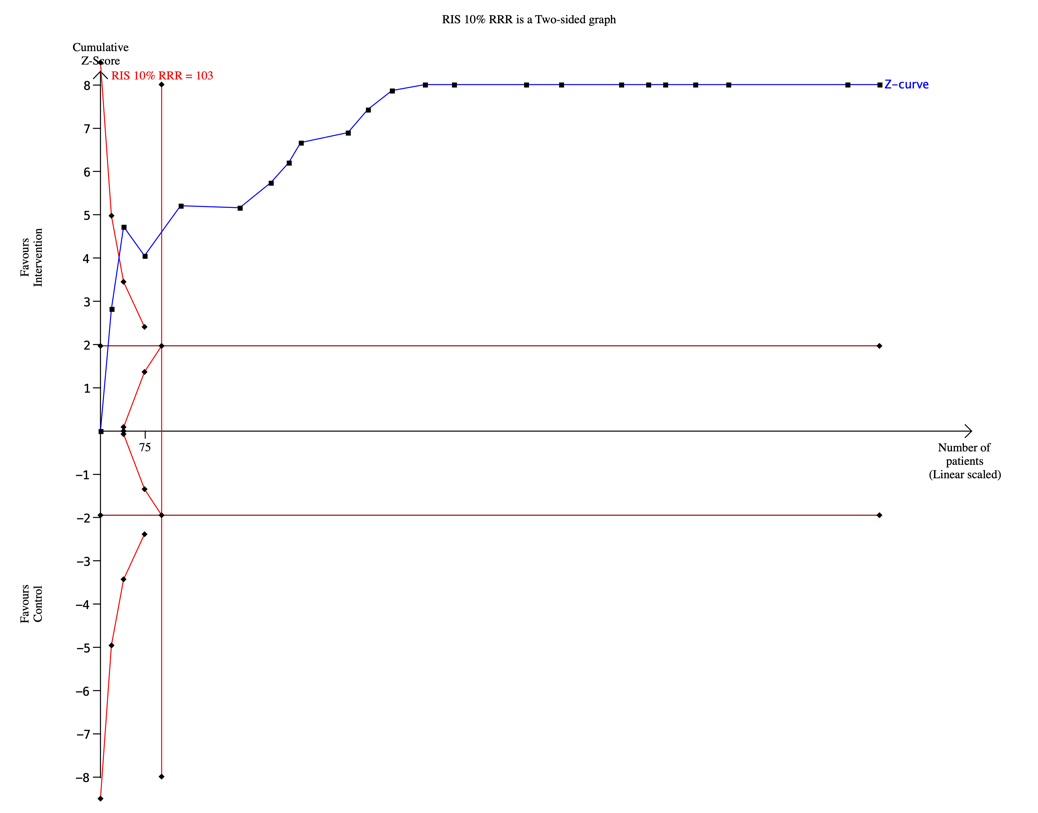

Supplement: Multimedia component 1 [file mmc1.docx]
